# Mechanistic Understanding of Pandemic Dynamics: A Multiscale Algorithmic Framework

**DOI:** 10.3390/life16060889

**Published:** 2026-05-25

**Authors:** Dimitris M. Manias, Dimitrios G. Patsatzis, Haralampos Hatzikirou, Dimitris A. Goussis

**Affiliations:** 1 Mathematics Department, Khalifa University of Science and Technology, Abu Dhabi 127788, United Arab Emirates; dimitris.manias@ku.ac.ae (D.M.M.); haralampos.hatzikirou@ku.ac.ae (H.H.); 2 Modelling Engineering Risk & Complexity, Scuola Superiore Meridionale, 80125 Napoli, Italy; d.patsatzis@ssmeridionale.it; 3 Biotechnology Center, Khalifa University of Science and Technology, Abu Dhabi 127788, United Arab Emirates; 4 Center for Information Services and High Performance Computing, Technische Universitat Dresden, 01062 Dresden, Germany; 5 Department of Mechanical Engineering, Khalifa University of Science and Technology, Abu Dhabi 127788, United Arab Emirates

**Keywords:** predictive models of pandemics, COVID-19, population dynamics, time scale analysis, computational singular perturbation

## Abstract

We present a robust, data-efficient framework for early outbreak assessment using multiscale analysis and Computational Singular Perturbation (CSP). This framework overcomes the shortcomings of the standard compartmental epidemiological models, which often struggle with parameter identifiability during the early stages of a pandemic, limiting their predictive utility considerably when data is sparse. Rather than relying on curve-fitting population profiles, which are sensitive to uncertainty, our approach isolates the dominant “explosive time scale that characterizes the outbreak’s intensity and duration. Using a calibrated SEIRD model, CSP allows for the identification of the paths that drive the process during the outbreak phase and the critical transition from accelerating to decelerating growth, which serves as a reliable precursor to the epidemic peak. This framework is assessed against the 4th, 5th, and 6th waves of the COVID-19 pandemic in Greece during 2021, covering periods dominated by the Delta and Omicron variants. Using only early-stage data from short calibration windows, CSP diagnostic tools revealed distinct dynamical drivers for each wave; e.g., a transition from the 4th wave that was driven by transmission intensity (Delta variant dominance) to the 6th wave that was driven by rapid exposure-to-infection turnover and reduced opposition from recovery mechanisms (Omicron variant dominance). Furthermore, it is demonstrated that the timing of the outbreak’s weakening can be accurately predicted, demonstrating robustness with results obtained from longer observation windows. These findings position multiscale analysis as a powerful, pathogen-agnostic early-warning system, capable of disentangling complex epidemic mechanisms and assessing intervention efficacy in real-time.

## 1. Introduction

Pandemics have historically posed critical challenges to global public health, prompting extensive efforts across the mathematical and computational sciences to model, understand, and predict their progression. The COVID-19 pandemic, which began in late 2019, intensified this research, with early studies focusing on characterizing the initial outbreak dynamics [1,2,3], performing comparative analyses across countries [4,5,6], and examining spatial transmission patterns within national and regional settings [7,8]. As the pandemic progressed, attention focused toward quantifying the effectiveness of non-pharmaceutical interventions [9,10,11], mitigating healthcare system overload [12,13,14], and designing optimal exit strategies [15,16,17]. Throughout all stages, predictive modeling of epidemic trajectories and real-time estimation of the reproduction number remained central objectives [18,19,20].

This sustained research effort has produced a wide array of mathematical modeling approaches. Ordinary differential equation (ODE) compartmental models remain foundational [21,22,23,24], complemented by agent-based models [6,25,26] and meta-population formulations [27,28,29]. In parallel, data-driven approaches including machine learning [30,31], deep learning [32,33,34], Bayesian inference, and autoregressive time-series techniques [35,36,37] have been increasingly adopted. Systematic reviews of early COVID-19 forecasting efforts highlight both the breadth of methodologies employed and the substantial uncertainty associated with early predictions [38,39,40].

Among these approaches, compartmental epidemic models remain the most widely used due to their analytical tractability, interpretability, and ability to incorporate mechanistic understanding of disease transmission. The classical SIR model [41,42] and its extensions can capture key epidemiological characteristics [43,44], including presymptomatic transmission, asymptomatic infections, and disease-induced mortality [45,46]. For COVID-19, SEIRD-type models were shown to be particularly appropriate due to the presence of an incubation period and non-negligible fatality rates [47,48,49,50]. The SEIRD model has been extended to incorporate vaccination dynamics and intervention measures in order to improve epidemic prediction under evolving public-health conditions [51]. Comparative studies demonstrated that increasing model complexity does not necessarily lead to improved early predictive performance, especially under limited data availability [52].

Early forecasting based on compartmental models demonstrated both their utility and their limitations. While short-term predictions captured initial exponential growth, longer-term forecasts were often highly sensitive to parameter uncertainty and structural assumptions [53]. Parameter identifiability issues are particularly severe during the early outbreak phase, where sparse, delayed, and heterogeneous data lead to multiple parameter sets that fit observations equally well but diverge significantly in their predictions [54,55,56]. These challenges have been documented extensively for COVID-19 [38,57,58]. Hybrid approaches combining mechanistic models with data-driven methods have been proposed to mitigate these issues, though they remained sensitive to reporting delays, intervention changes, and calibration strategies [44,59,60,61,62].

A particularly influential line of work has focused on identifying early dynamical signatures of epidemic transitions. Change-point detection and Bayesian inference techniques have revealed how intervention measures alter transmission dynamics during the early exponential phase [40]. Early warning systems were designed to predict epidemic surges or peaks before they show up in official case data [63,64]. Nevertheless, many of these methods still rely heavily on sufficient observational data and may struggle precisely when early prediction is most critical.

Motivated by the limitations of existing early prediction approaches, and particularly their reliance on extensive parameter calibration and large, high-quality datasets, this work proposes an alternative framework for early pandemic outbreak prediction grounded in time scale analysis of epidemic dynamics. Rather than focusing on solution profiles, which are often highly sensitive to parameter non-identifiability, the proposed approach exploits the intrinsic multiscale structure of compartmental epidemic models. Epidemic systems naturally exhibit processes evolving over multiple time scales, encompassing interactions between transmission, latency, recovery, and population turnover mechanisms [65,66]. By identifying and analyzing the time scales characterizing these interactions, it is possible to infer critical features of outbreak evolution, such as the driving paths, the critical population groups or regime shifts, using limited early-phase data. This time scale–based perspective provides a complementary and data-efficient pathway for early outbreak prediction, strengthening the theoretical foundations of epidemic forecasting and aligning with broader calls for system-oriented modeling frameworks capable of integrating complex epidemic dynamics [34,67].

Herein, the use of CSP [68,69] is proposed as the core methodological tool for implementing this time scale–based framework in compartmental epidemic models. CSP is an algorithmic method of Geometrical Singular Perturbation Analysis (GSPA), designed for the analysis of multiscale dynamical systems that are driven by processes evolving over widely separated fast and slow time scales. In such systems, processes associated with fast time scales are rapidly equilibrated, leading to the formation of low-dimensional structures in the system’s tangent space, known as Slow Invariant Manifolds (SIMs) [70]. System trajectories subsequently evolve along these manifolds, governed by the remaining slow processes. CSP provides a complete and systematic set of diagnostic tools for identifying (i) the variables and processes associated with fast time scales that generate the SIM and (ii) the slow processes that dominate the long-term system dynamics [71]. Important CSP terminology is listed in Table 1.

Originally developed for the analysis of chemical kinetic mechanisms [72,73], CSP has since emerged as a general framework for multiscale dynamical systems, with applications spanning systems biology [74,75], pharmacokinetics [76,77], brain lactate metabolism [78,79], glycolysis [80], and population dynamics [81].

To demonstrate the systems-level understanding that can be acquired with the proposed CSP-based time scale analysis framework, we apply it to the case of the COVID-19 epidemic in Greece. The analysis will focus on the dynamics of the outbreak of the epidemic wave. Given prior knowledge regarding the existence of a latency period following infection with the SARS-CoV-2 virus [47,48], a SEIRD model is employed, incorporating exposed and deceased population groups. Specifically, we examine the 4th, 5th, and 6th epidemic waves in Greece, which occurred in July 2021, October 2021, and December 2021, respectively. Each wave was driven by different underlying dynamics, reflecting changes in transmission intensity, population behavior, and intervention policies. Using CSP diagnostic tools, we focus on the mode that drives the outbreak, and we identify the dominant populations and transition processes governing the outbreak phase of each epidemic wave, based solely on early-stage dynamics and limited observational data. This case study illustrates the method’s ability to disentangle evolving epidemiological mechanisms and highlights its potential as a robust early-warning tool for pandemic outbreak dynamics.

**Figure 1:**
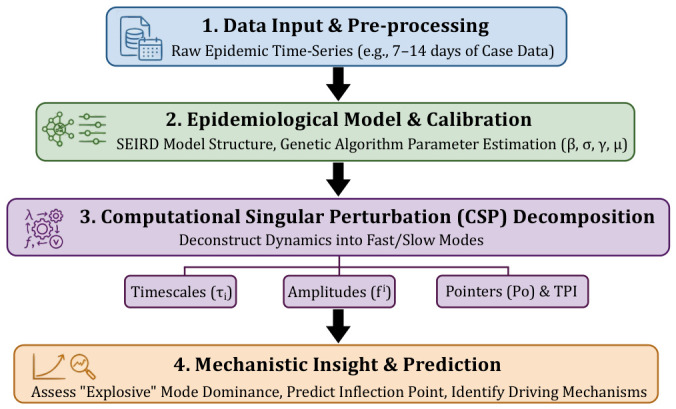
Methodological flowchart of the proposed early-warning framework. Early outbreak data are first fitted to the SEIRD model. CSP analysis is then applied to compute the characteristic time scales of the outbreak and the CSP diagnostics.

The overall methodological framework adopted in this study is summarized in Figure 1. The flowchart illustrates the sequential procedure from early outbreak data acquisition to CSP-based dynamical analysis and inflection point prediction.

## 2. Materials and Methods

### 2.1. The Mathematical Model

**Figure 2:**
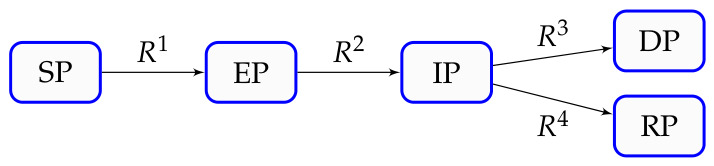
The SEIRD population model employed; SP: susceptible population (not infected), EP: exposed population (infected but not yet infectious), IP: infected population (able to transmit the virus), DP: deceased population, RP: recovered population. $R^{k}$ denotes the transmission rate of the *k*-th path.

According to the SEIRD model, the population of susceptible individuals (SP) might become exposed ones (EP) upon transmission of the virus; i.e., individuals that are infected but not yet infectious. After the incubation period, the exposed individuals become infected (IP), so they are able to transmit the virus. The infected individual can then either become deceased (DP) or recovered (RP). The paths that connect the five population groups, according to the SEIRD model, are depicted schematically in Figure 2.

The governing equations of the SEIRD model are

(1) $$\frac{d}{dt}\left[\begin{array}{c} SP\\ EP\\ IP\\ RP\\ DP \end{array}\right]=\left[\begin{array}{c} -\beta IP\;SP\\ \beta IP\;SP-\sigma EP\\ \sigma EP-(\gamma +\mu )IP\\ \gamma IP\\ \mu IP \end{array}\right],$$

where β is the transmission density-dependent ratio, σ is the transition ratio from exposed to infected individuals, expressing the inverse of the incubation period of the disease, γ is the recovery ratio, which also expresses the inverse of the infection period of the disease, and μ is the fatality ratio. Note that SP, EP and IP are decoupled from RP and DP. The SEIRD model in Equation (1) can be cast in the form

(2) $$\frac{dy}{dt}=S_{1}R^{1}+S_{2}R^{2}+S_{3}R^{3}+S_{4}R^{4}=g\left(y\right),$$

where y is the 5-dimensional column *state vector*, the elements of which represent the number of individuals in the population groups; $y={\left[\begin{array}{c} SP,EP,IP,RP,DP \end{array}\right]}^{⊤}$. The 5-dimensional column *vector field* g(y) incorporates the transition rates from one compartmental group to another. The column vectors $S_{k}$ (*k* = 1,…,4) denote the directions of the 4 transitions from a population group to another; $S_{1}={\left[\begin{array}{c} -1,1,0,0,0 \end{array}\right]}^{⊤}$, $S_{2}={\left[\begin{array}{c} 0,-1,1,0,0 \end{array}\right]}^{⊤}$, $S_{3}={\left[\begin{array}{c} 0,0,-1,0,1 \end{array}\right]}^{⊤}$, $S_{4}={\left[\begin{array}{c} 0,0,-1,1,0 \end{array}\right]}^{⊤}$. The scalars $R^{k}$ (*k* = 1,…,4) are related to $S_{k}$ rates of transition; $R^{1}=\beta IP\;SP$, $R^{2}=\sigma EP$, $R^{3}=\mu IP$ and $R^{4}=\gamma IP$, which denote the transmission, incubation, recovery and death rates, respectively. The model in Equation (2) is subject to two conservation laws

(3) $$\begin{array}{c} \frac{d(SP+EP+IP+RP+DP)}{dt}=0\;\mathrm{and}\;\;\frac{d(\gamma DP-\mu RP)}{dt}=0. \end{array}$$

which indicate that the process evolves in a 3-dimensional subspace of the 5-dimensional phase space.

### 2.2. Local Stability Analysis

The existence of the conservation laws in Equation (3) implies that the dynamical evolution of the system is restricted to a 3-dimensional manifold. To characterize the local stability within this subspace, we analyze the Jacobian matrix J associated with the vector field in Equation (2), given by

(4) $$J=\left[\begin{array}{ccccc} -\beta IP & 0 & -\beta SP & 0 & 0\\ \beta IP & -\sigma & \beta SP & 0 & 0\\ 0 & \sigma & -(\gamma +\mu ) & 0 & 0\\ 0 & 0 & \gamma & 0 & 0\\ 0 & 0 & \mu & 0 & 0 \end{array}\right].$$

The characteristic equation det(J−λI)=0 yields two eigenvalues that are identically zero, $\lambda_{4,5}=0$, which directly correspond to the two conservation laws of Equation (3). The remaining three eigenvalues are the roots of the characteristic polynomial

(5) $$P\left(\lambda \right)=\lambda^{3}+a_{2}\lambda^{2}+a_{1}\lambda +a_{0}=0,$$

which are given as

(6) $$\lambda_{n}=-\frac{a_{2}}{3}+\omega^{n}\sqrt[{3}]{-\frac{q}{2}+\sqrt{\frac{q^{2}}{4}+\frac{p^{3}}{27}}}+\omega^{2n}\sqrt[{3}]{-\frac{q}{2}-\sqrt{\frac{q^{2}}{4}+\frac{p^{3}}{27}}},$$

for n=1,…,3, where

$$p=\frac{3a_{1}-a_{2}^{2}}{3},\;q=\frac{2a_{2}^{3}-9a_{2}a_{1}+27a_{0}}{27},\;\omega =e^{i\frac{2\pi }{3}}$$

$$a_{2}=\beta IP+\sigma +\gamma +\mu ,\;a_{1}=\beta IP\left(\sigma +\gamma +\mu \right)+\sigma \left(\gamma +\mu \right)-\sigma \beta SP,\;a_{0}=\beta IP\sigma \left(\gamma +\mu \right).$$

These expressions provide the local spectral properties of the system at any point within the feasible region of the phase space.

### 2.3. Model Calibration

The model was calibrated against the daily cases of confirmed infected (IP), recovered (RP) and deceased (DP) individuals, as reported by the Greek government COVID-19 data repository provided by iMEdD-Lab [82]. The initial conditions of the susceptible (SP) and exposed (EP) individuals and the parameter values of β, σ, μ and γ were estimated using the Genetic algorithm [83] provided by COPASI [84], on the basis of the reported data sets of IP, RP and DP. Additional details regarding the COPASI-based calibration procedure, optimization settings, parameter bounds, and fitted quantities for all epidemic waves are provided in Appendix A. Since this study focuses on the outbreak phase of three different epidemic waves, the model was calibrated for two periods of each wave in order to demonstrate the robustness of the results. The starting point of both periods was the same (the start of the wave), while the end of these periods was different, but within the outbreak phase; see Section 3.1, Section 3.2 and Section 3.3 for details. The parameter values thus estimated for all six periods are displayed in Table 2.

### 2.4. Modeling Assumptions and Vaccination Effects

It is noted that during the three epidemic waves considered here, vaccination coverage constituted an important factor. The vaccination campaign expanded during the first half of 2021, starting from older populations in late January 2021 and reaching 30–59 years old in April of that year [85]. Therefore, vaccination had no important effect during the 4th wave (July 2021), but had an influence during the 5th and 6th waves (October–November 2021 and December 2021–January 2022, respectively); although, due to the prevailing Omicron new variant during the 6th wave [86,87], the vaccine might have been not as effective as it was with the Delta variant that prevailed during the 4th and 5th waves [88]. The SEIRD framework employed here does not explicitly include dedicated compartments for vaccinated individuals or waning immunity (i.e., reinfections). This modeling choice was made to preserve structural parsimony and to focus on the dynamical properties of the outbreak phase rather than on detailed population stratification.

Importantly, the CSP-based time scale analysis relies on the local dynamical structure of the system, as encoded in the Jacobian matrix and in the data of the fitting window considered. Consequently, the net effects of vaccination, partial immunity, and reinfection are implicitly reflected in the generated fitted model. Variations in susceptibility, reduced infectiousness, or accelerated recovery due to vaccination or reinfection during the epidemic wave manifest in the generated model, which in turn determines the characteristic dynamics.

### 2.5. Time Scale Analysis and CSP Tools

In the CSP context, Equation (2) is cast in the form

(7) $$\begin{array}{c} \frac{dy}{dt}=\sum_{n=1}^{5}a_{n}f^{n}\;\mathrm{and}\;f^{n}=\sum_{m=1}^{4}\left(b^{n}\cdot S_{m}\right)R^{m} \end{array},$$

where the column CSP vectors $a_{n}$ (*n* = 1,…,5) span the 5-dimensional tangent space, the row vectors $b^{n}$ are their dual ($b^{i}\cdot a_{j}=\delta_{j}^{i}$) and the amplitudes $f^{n}$ are the projections of the vector field g(y) on $a_{n}$ (by proper adjustment of the signs of the CSP basis vectors) [68,69]. Each mode $a_{n}f^{n}$ associates with a time scale $\tau_{n}$, ordered so that $\tau_{1}<\tau_{2}<\ldots$. The amplitude $f^{n}$ provides a measure of the impact of the mode, while the time scale $\tau_{n}$ provides a measure of the time frame of its action. The time scale $\tau_{n}$ can be explosive or dissipative, depending on whether the components of the vector field responsible for its generation tend to move the trajectory away or towards equilibrium [89,90]. Due to the conservation laws in Equation (3), $f^{4}=f^{5}=0$ and $\tau_{4}=\tau_{5}=\infty$. As a result, only the first three modes will be retained in Equation (7) and the last two will be neglected. According to CSP, the basis vectors $a_{n}$ are computed via a refinement procedure that provides, order by order, the higher order corrections in the context of asymptotic analysis [91,92].

It will be shown next that during the outbreak phase of all three pandemic waves considered, the fastest time scale $\tau_{1}$ is of dissipative character (henceforth, $\tau_{1}=\tau_{d}$ and $f^{1}=f^{d}$), while the other two time scales are of explosive one (henceforth, $\tau_{2}=\tau_{e,f}$, $\tau_{3}=\tau_{e,s}$ and $f^{2}=f^{e,f}$, $f^{3}=f^{e,s}$); where $\tau_{d}<\tau_{e,f}<\tau_{e,s}$. Due to the dissipative character of the fastest time scale $\tau_{d}$, the magnitude of the related amplitude $f^{d}$ is the outcome of significant cancellations among the additive terms in the rhs of its expression

(8) $$\begin{array}{c} f^{d}=\left(b^{d}\cdot S_{1}\right)R^{1}+\left(b^{d}\cdot S_{2}\right)R^{2}+\left(b^{d}\cdot S_{3}\right)R^{3}+\left(b^{d}\cdot S_{4}\right)R^{4}; \end{array}$$

i.e., $f^{d}/max\left|\left(b^{d}\cdot S_{k}\right)R^{k}\right|\ll 1$, where the magnitude of this ratio is proportional to the ratio $\tau_{d}/\tau_{e,f}$ [69,91]. As it will be shown next, when the pandemic waves will be analyzed, these cancellations tend to keep the amplitude $f^{d}$ small, so that the process is mainly driven by the dominant of the two slower explosive modes; see Appendix B for details. Interested in leading order accuracy, the CSP basis vectors can be represented by the right eigenvectors of the Jacobian J(y) of g(y); i.e., $a_{n}=\alpha_{n}$ and $b^{n}=\beta^{n}$, where $\alpha_{n}$ and $\beta^{n}$ are the right and left eigenvectors of J(y), satisfying $\beta^{i}\cdot \alpha_{j}=\delta_{j}^{i}$ [68]. Therefore, the CSP vectors rotate in phase space as the process evolves. The CSP formulation in the form of Equation (7) allows the development of various diagnostics tools, among them the *Pointer* (*Po*) and the *Time scale Participation Index* (*TPI*).

Each population group is associated with each CSP mode with varying intensity. The degree to which each group associates with the *n*-th CSP mode (n=1,2,3) is quantified by the *Pointer* (*Po*) metric

(9) $$D^{n}=\mathrm{diag}\left[\alpha_{n}\beta^{n}\right]=\left[\beta_{1}^{n}\alpha_{n}^{1},\;\beta_{2}^{n}\alpha_{n}^{2},\;\beta_{3}^{n}\alpha_{n}^{3},\;\beta_{4}^{n}\alpha_{n}^{4},\;\beta_{5}^{n}\alpha_{n}^{5}\right],$$

where, due to the orthogonality of the CSP basis vectors, the following relation holds $\sum_{i=1}^{5}\beta_{i}^{n}\alpha_{n}^{i}=1$ [93]. The components of $D^{n}$ assess the way the four populations will adjust when the system is perturbed along the direction of the basis vector $\alpha_{n}$ [94].

The time scales will be approximated by the inverse of the eigenvalues of the Jacobian J(y) of Equation (6); i.e., $\tau_{n}={\left|\lambda_{n}\right|}^{-1}$ (n=1,2,3) [89]. This formulation allows the introduction of the *Time scale Participation Index* (*TPI*) as

(10) $$J_{k}^{n}=\frac{c_{k}^{n}}{\sum_{i=1}^{4}\left|c_{i}^{n}\right|},\;\;c_{k}^{n}=\beta^{n}\nabla \left(S_{k}R^{k}\right)\alpha_{n},\;\;\lambda_{n}=c_{1}^{n}+\ldots +c_{4}^{n},$$

where k=1,…,4, n=1,…,3 and by definition $\sum_{k=1}^{4}\left|J_{k}^{n}\right|=1$ [95,96]. The term $c_{k}^{n}$ denotes the contribution of the *k*-th path to the *n*-th time scale and can be either positive or negative. $J_{k}^{n}$ identifies the paths in the SEIRD model that contribute significantly to the generation of the time scale $\tau_{n}$, while negative (positive) $J_{k}^{n}$ implies that the *k*-th path contributes to a dissipative (explosive) character of the *n*-th time scale $\tau_{n}$.

In the case of a complex pair of eigenvectors, where $\alpha_{k}=\alpha_{r}+i\alpha_{i}$ and $\alpha_{k+1}=\alpha_{r}-i\alpha_{i}$ ($\lambda_{k}=\lambda_{r}+i\lambda_{i}$ and $\lambda_{k+1}=\lambda_{r}-i\lambda_{i}$), the related pair of basis vectors is defined as $a_{k}=\alpha_{r}$ and $a_{k+1}=\alpha_{i}$. In that case, the quantities ($f^{k}$, $D^{k}$) will refer to the real part ($\alpha_{r}$), and the quantities ($f^{k+1}$, $D^{k+1}$) will refer to the imaginary part ($\alpha_{i}$) [95].

## 3. Results

The dynamics of the 4th, 5th and 6th waves of the COVID-19 epidemics in Greece that will be reported here were obtained according to the procedure described in Figure 1. In order to demonstrate robustness and consistency of the results, two fitting windows are considered for each wave, having the same starting point.

### 3.1. The 4th Wave

The analysis of the 4th COVID-19 wave was conducted using solutions of the SEIRD model, which was fitted to data from the following two time periods:

(i) Period A: July 1–July 8 (8 days);
(ii) Period B: July 1–July 18 (18 days).

July 1 is considered the onset of the wave. It is noted that the Greek government introduced restriction measures on July 16, near the end of Period B [97].

**Figure 3:**
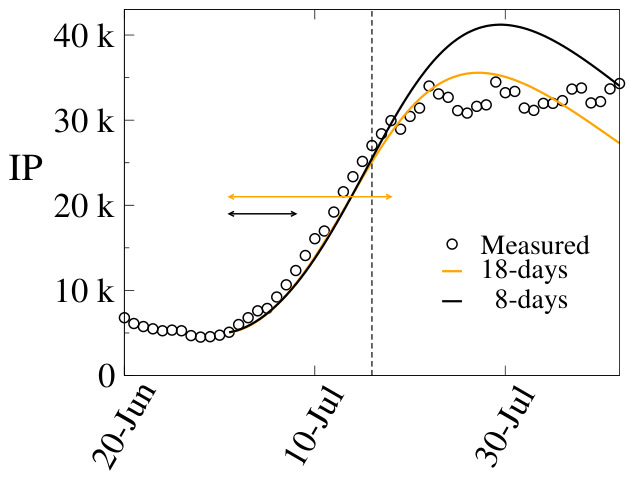
Fourth wave. Active cases (IP, see Figure 2) based on official data (circles) and SEIRD model simulations (curves) fitted to Period A (July 1–8) and Period B (July 1–18). Horizontal arrows denote the two periods, and the vertical dashed line marks the date of government-imposed restrictions on July 16 [97].

Figure 3 displays the active cases (IP) from the COVID-19 data repository [82] (shown as circles) up to 12 August 2021, along with the predictions from the SEIRD, which was calibrated to Periods A (8 days) and B (18 days). In both cases, the model captures with significant accuracy the evolution of active cases during the fitting window.

**Figure 4:**
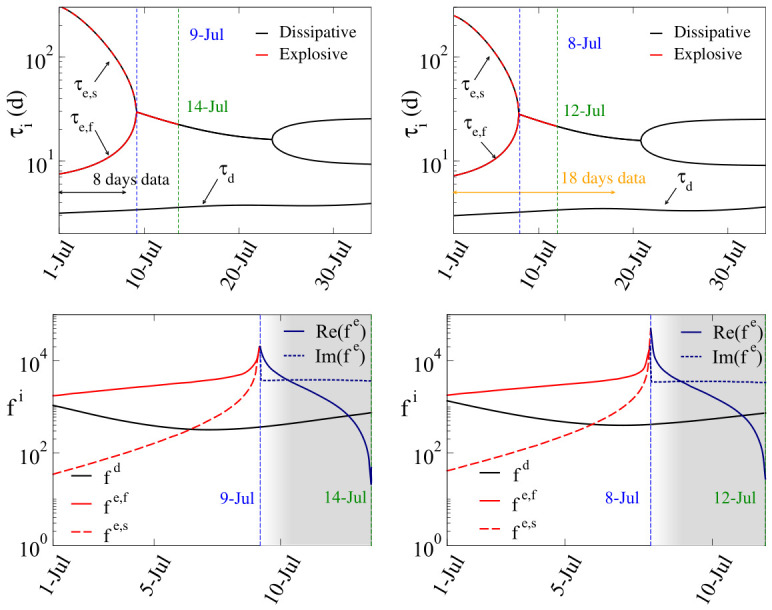
Fourth wave. (**Top**): Explosive (red) and dissipative (black) time scales. (**Bottom**): Amplitudes of explosive (red) and dissipative (black) modes. (**Left**): model fitted to 8-day data (Period A); (**Right**): fitted to 18-day data (Period B). Vertical blue dashed line: merge of explosive time scales; vertical green dashed line: disappearance of explosive dynamics. Bottom row gray region: amplitudes that refer to the real (solid) and imaginary (dashed) parts in the case of complex-conjugate explosive eigenvalues.

The outbreak dynamics during the 4th wave exhibit one fast *dissipative* time scale (characterizes the action of a mode that tends to attenuate the outbreak), $\tau_{d}$, and two slow *explosive* time scales (characterize the action of two modes that tend to amplify the outbreak); one faster, $\tau_{e,f}$, and one slower, $\tau_{e,s}$. The temporal evolution of the time scales is displayed in the top panels of Figure 4, while the amplitudes of the corresponding modes ($f^{d}$, $f^{e,f}$, and $f^{e,s}$) are displayed in the bottom panels. A comparison of the profiles obtained on the basis of the two fitting periods indicates that the findings are quite robust. During the initial part of the outbreak on July 1, the two explosive time scales $\tau_{e,f}$ and $\tau_{e,s}$ are distinct, but progressively they approach each other. During this period, the amplitude of the fastest explosive time scale indicates that the related CSP mode $a_{e,f}f^{e,f}$ dominates in driving the process. Eventually, the two time scales merge into a single explosive time scale (on July 9 for Period A and July 8 for Period B), which subsequently evolves into a dissipative time scale (on July 14 for Period A and July 12 for Period B) and then into two distinct dissipative ones, as shown in the top panels of Figure 4. This is a typical process whereby two real positive eigenvalues of the system’s Jacobian evolve into two real negative. During this process, the real part changes sign, while the imaginary part does not vary much and dominates the characteristic time scale there. Therefore, in the following, the term “*explosive stage*” will refer to the part of the outbreak in which the two explosive time scales are distinct. It is noted that the merging of the two time scales $\tau_{e,f}$ and $\tau_{e,s}$ is accompanied by the merging of two amplitudes $f^{e,f}$ and $f^{e,s}$.

Given the focus of this study on the outbreak dynamics, the origins of the fastest explosive time scale $\tau_{e,f}$ will be examined, since the related mode $a_{e,f}f^{e,f}$ is the one driving the process during the explosive stage. Using the Time scale Participation Index (TPI) defined in Equation (10), Table 3 quantifies the major contributions to $\tau_{e,f}$ from the 4 paths in the model, as computed at the start of the 4th wave (July 1) and near the end of the explosive stage (July 7). It is demonstrated that the models based on both A and B periods provide qualitatively similar results. It is also shown that the major contributors to the explosive character of $\tau_{e,f}$ are Path 1 (SP → EP) followed by Path 2 (EP → IP), while Path 4 (IP → RP) exerts the major opposition; Path 3 (IP→DP) exhibits negligible contribution. Table 3 also shows that the promoting influence of Paths 1 and 2 decreases with time, while the opposing effect of Path 4 approximately doubles.

Using the Pointer index (Po) that was defined in Equation (9), Table 3 lists the population groups associated the most with the fastest explosive mode. Similarly to the TPIs, it is demonstrated that the Pos computed on the basis of the A and B periods provide qualitatively similar results. It is shown that the fastest explosive mode tends to increase the IP and EP populations and decrease the SP population. All these trends are shown to intensify with time. It is noted that the consistency of the conclusions drawn using the available data of the two periods underscores the robustness of the proposed methodology, even when only limited early-stage data are available.

### 3.2. The 5th Wave

The dynamics of the 5th COVID-19 wave were analyzed by fitting the SEIRD model to the data from the following two periods:

(i) Period C: October 10–October 23 (14 days);
(ii) Period D: October 10–November 6 (28 days).

October 10 was considered the start of the 5th wave. Notably, restriction measures were implemented on November 6, coinciding with the end of Period D [98].

**Figure 5:**
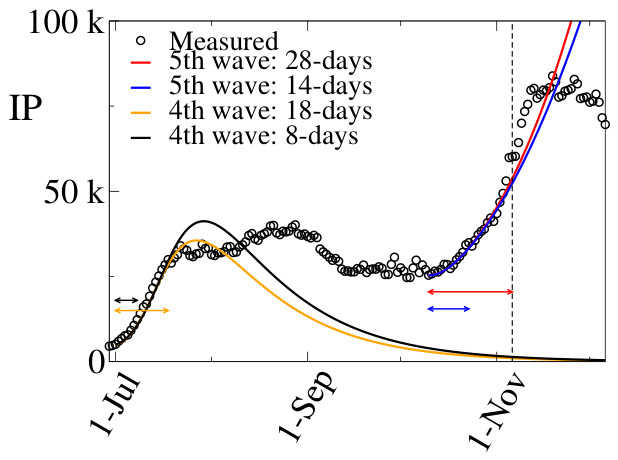
Fifth wave. Active cases (IP) based on official data (circles) and SEIRD model predictions (curves), calibrated using data from Periods C (October 10–23) and D (October 10–November 6). Horizontal arrows indicate the data-fitting intervals. For comparison, model outputs based on Periods A and B are also shown. The vertical dashed line marks the date of governmental restrictions on November 10 [98].

Figure 5 displays the evolution of active cases (IP), as reported in the Greek COVID-19 data repository [82], along with the SEIRD model outputs fitted to data from Periods C and D. In both cases, the model reproduces the active case profile within the calibration window with significant accuracy. For comparison, extended model outputs based on fittings from Periods A and B (related to the 4th wave) are also shown.

**Figure 6:**
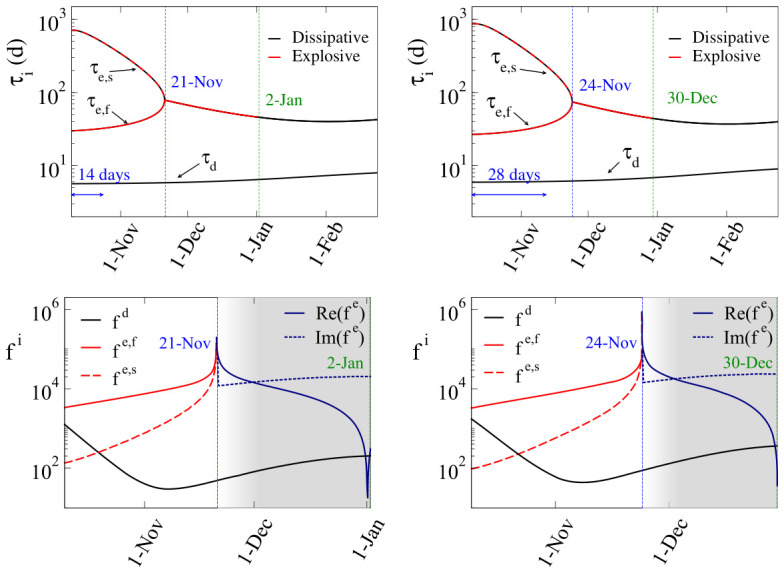
Fifth wave. (**Top**): explosive (red) and dissipative (black) time scales. (**Bottom**): corresponding amplitudes for explosive (red) and dissipative (black) modes, based on data from Period C (14 days, (**left**)) and Period D (28 days, (**right**)). The blue dashed line indicates the coalescence of the explosive time scales, while the green dashed line marks the disappearance of explosive dynamics. Bottom row gray region: amplitudes that refer to the real (solid) and imaginary (dashed) parts in the case of complex-conjugate explosive eigenvalues.

As in the 4th wave, the outbreak dynamics is characterized by one fast *dissipative* time scale $\tau_{d}$ (characterize the action of a mode that tends to attenuate the outbreak) and two slow *explosive* time scales (characterize the action of two modes that tends to amplify the outbreak), a faster $\tau_{e,f}$ and a slower one $\tau_{e,s}$. Their temporal evolution is shown in the top panels of Figure 6, while the profiles of the corresponding amplitudes ($f^{d}$, $f^{e,f}$, $f^{e,s}$) are presented in the bottom panels. Once more, it is demonstrated that both Periods C and D produce qualitatively similar profiles. Similarly to the 4th wave, the two explosive time scales are distinct at the start of the 5th wave (October 10) and progressively approach each other. Eventually, the two time scales merge on November 21 for Period C and November 24 for Period D, which mark the end of the explosive stage. Again, Figure 6 shows that the mode $a_{e,f}f^{e,f}$ relating to the fastest explosive time scale $\tau_{e,f}$ is shown to drive the process during this stage.

The paths that contribute the most to the explosive time scale $\tau_{e,f}$ and the populations that related the most to the explosive mode $a_{e,f}f^{e,f}$ are listed in Table 4. The displayed results refer to the start of the 5th wave (October 10) and to a point near the end of the explosive stage (November 20). As in the 4th wave, it is shown that (i) the explosive character of $\tau_{e,f}$ is mainly promoted by Paths 1 and 2 (SP → EP and EP → IP) and opposed by Path 4 (IP → RP) and (ii) the promoting contributions of Paths 1 and 2 decrease with progressing time and that of the opposing contribution of Path 4 increases. While in the case of the 4th wave, the promoting contributions of Paths 1 and 2 were the largest in magnitude, followed by the opposing one of Path 4, Table 4 shows that in the case of the 5th wave, the magnitude of the opposing contribution of Path 4 is ranked second, overtaking that of the promoting contribution of Path 2.

Regarding the populations related to the fastest explosive mode, the conclusions drawn from Table 4 are similar to those drawn from Table 3; i.e., the mode tends to increase that IP and EP populations and decrease the SP population, and that these trends intensify with progressing time. Once more, it is noted that the conclusions reached on the basis of the available data in Periods C and D were qualitatively similar.

### 3.3. The 6th Wave

The dynamics of the 6th COVID-19 wave were based on the data of the following two periods:

(i) Period E: December 26–January 1 (7 days);
(ii) Period F: December 26–January 4 (10 days).

December 26 is considered the beginning of the 6th wave. It is noted that social restrictions were implemented on December 30 [99].

**Figure 7:**
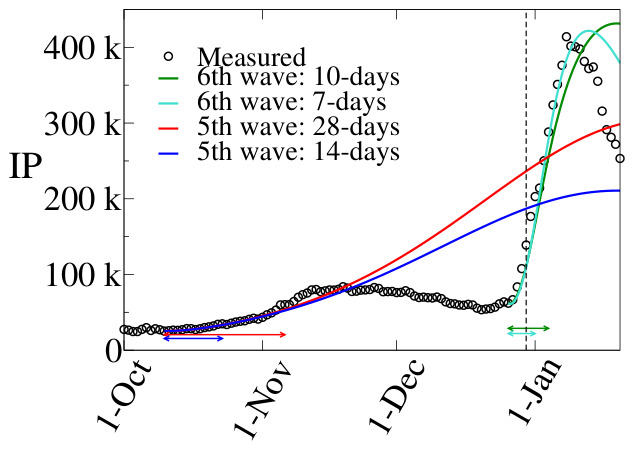
Sixth wave. Active cases (IP) based on official data (circles) and SEIRD model predictions (curves), fitted to data from Periods E (Dec 26–Jan 1) and F (Dec 26–Jan 4). For comparison, model outputs from Periods C and D are also shown. Horizontal arrows indicate the fitting intervals. The vertical dashed line marks the date when social restrictions were enacted [99].

Figure 7 illustrates the evolution of active cases (IP) as reported by the Greek COVID-19 data repository [82], along with SEIRD model outputs calibrated to Periods E and F. In both cases, the model accurately captures the observed trend within the respective fitting windows. Extended predictions based on data from Periods C and D are also displayed for comparison.

**Figure 8:**
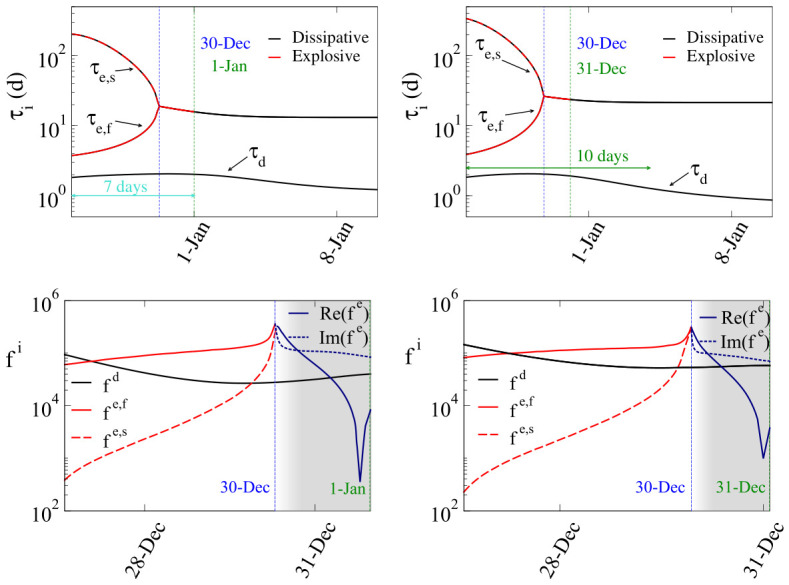
6th wave. (**Top**): explosive (red) and dissipative (black) time scales. (**Bottom**): amplitudes of the explosive (red) and dissipative (black) modes, based on fitting data from Period E (**left**) and Period F (**right**). The blue dashed line indicates the coalescence of the explosive time scales, while the green dashed line marks the disappearance of explosive dynamics. Bottom row gray region: amplitudes that refer to the real (solid) and imaginary (dashed) parts in the case of complex-conjugate explosive eigenvalues.

As in the previous cases examined, the outbreak dynamics during the 6th wave are characterized by one fast dissipative time scale $\tau_{d}$ (characterize the action of a mode that tends to attenuate the outbreak) and two slow explosive time scales (characterize the action of two modes that tends to amplify the outbreak); a faster $\tau_{e,f}$ and a slower one $\tau_{e,s}$. The top panels of Figure 8 display the profiles of the three time scales, while the bottom panels display the corresponding amplitudes ($f^{d}$, $f^{e,f}$, $f^{e,s}$).

Figure 8 shows that the explosive stage spans the period from December 26 to December 30 for Period E and to January 1 for Period F, at which point the two explosive time scales meet. As with the previous two cases, the amplitude profiles in Figure 8 indicate that the mode $a_{e,f}f^{e,f}$, which is characterized by the fastest explosive time scale $\tau_{e,f}$, dominates the evolution of the process in the outbreak stage, but not as much as in the case of the previous two waves.

Table 5 lists the major TPI and Po values of the fast explosive mode, defined in Equations (9) and (10), at the start of the 6th wave (December 26) and at a point near the end of the explosive stage (December 29). Once more, the two periods considered lead to qualitatively similar conclusions. In agreement with the previous cases, the TPI values listed indicate that the explosive character of $\tau_{e,f}$ is promoted by Paths 1 and 2 (SP → EP and EP → IP) and is opposed by Path 4 (IP → RP). In this case, the largest promoting contribution originates from Path 2, while the opposing contribution of Path 4 is very weak (significantly weaker when compared to the 4th and 5th waves). During the short period considered, the contribution of Path 2 remains practically constant, that of Path 1 decreases and that of Path 4 increases substantially. The Po values further show that the infected (IP) and exposed (EP) populations are the ones most associated with the fastest explosive mode $a_{e,f}f^{e,f}$, followed by the susceptible population (SP) throughout the explosive stage The association of IP and EP exhibits a moderate increase, while that of SP exhibits a significant increase.

As with the previous analyses, the diagnostics from Periods E and F are qualitatively consistent, demonstrating the reliability of the findings regarding the driving mechanisms of the 6th wave.

## 4. Physical Insights

The dynamical characteristics of the outbreak phase during the 4th, 5th, and 6th waves of the COVID-19 epidemic in Greece, as revealed with the CSP diagnostic tools, exhibit significant similarities and differences. These attributes are directly related to the degree to which each of the four paths in the model are shown to influence the outbreak progression. It will be demonstrated here that the CSP diagnostics can provide a system-level understanding of this process.

It was shown that in all three waves considered (i) the dynamics exhibited one fast dissipative time scale and two slow explosive ones, and (ii) the mode $a_{e,f}f^{e,f}$ that is characterized by the faster explosive time scale $\tau_{e,f}$ was the one driving the process during the outbreak initiation. Therefore, the magnitude of $\tau_{e,f}$ provides in all cases considered a measure of the outbreak intensity; i.e., a shorter/longer $\tau_{e,f}$ implies a faster/slower outbreak onset. The significance of $\tau_{e,f}$ is highlighted in Table 6, which lists its value at the start of each wave ($\tau_{e,f}^{0}$) and at the end of the explosive stage ($\tau_{e,f}^{*}$); the value of the fast dissipative time scale at the end of the explosive stage $\tau_{d}^{*}$ is also listed for comparison. As shown in Figure 4, Figure 6 and Figure 8, the time scale gap among the two fastest time scales, $\tau_{d}$ and $\tau_{e,f}$, increases along the explosive stage; reaching an O(10) difference in magnitude at the end of the stage, as shown in Table 6. Such an increasing time scale gap and the dissipative nature of the fastest time scale $\tau_{d}$ denote the progressing decoupling of the fast and slow dynamics [69,92]. This feature is manifested by the decrease of $f^{d}$ and the increase of $f^{e,f}$ along the explosive stage, which allows the mode $a_{e,f}f^{e,f}$ to dominate there.

The values of $\tau_{e,f}^{0}$ that are listed in Table 6 show that the fastest onset of the outbreak phase is recorded in the 6th wave, being about two times faster that that in the 4th wave and about seven times faster than in the 5th wave. Similar trends are also exhibited by the listed values of $\tau_{e,f}^{*}$. These differences in the intensity of the onset of the outbreak in the three cases considered reflect differences in the paths that were shown to contribute to the characteristic time scale $\tau_{e,f}$ during each wave.

This issue is addressed by the results displayed in Table 7, which summarizes the range of contributions of the various paths to $\tau_{e,f}$ during the explosive stage. For simplicity only the shortest Periods A, C and E are considered; qualitatively similar conclusions are reached by considering the longer Periods B, D and F. It is shown that in all three cases considered, Paths 1 and 2 (SP → EP and EP → IP) promote the explosive character of $\tau_{e,f}$, while Path 4 (IP → RP) opposes it; Path 3 (IP → DP) provides negligible contribution. It is also shown that the influence of Paths 1 and 2 weakens along the explosive stage, while that of Path 4 strengthens. The major contributor to $\tau_{e,f}$ during the faster onset, which is manifested in the 6th wave, is Path 2 (EP → IP), which is accompanied by the smallest opposing contribution from Path 4 (IP → RP). During the second-fastest onset, which is manifested in the 4th wave, the major contributor to $\tau_{e,f}$ is provided by Path 1 (SP → EP), while the opposing contribution from Path 4 (IP → RP) is the second-smallest in all three waves considered. Finally, during the slowest onset, which is manifested in the 5th wave, the major contributor to $\tau_{e,f}$ is provided by Path 1 (SP → EP) at similar levels as in the 4th wave, but the opposing contribution from Path 4 (IP → RP) is considerably larger than those in the 4th and 6th waves.

The main conclusion that can be drawn from the findings in Table 7 is that the intensity of the outbreak is shown to be directly related to the rates by which susceptible individuals become exposed and then infected, while the rate by which infected individuals recover tends to slow down the outbreak. This finding is reflected in the association of the various populations with the driving mode. In fact, Table 7 shows that the explosive mode $a_{e,f}f^{e,f}$ tends to increase IP and EP and decrease SP in all three waves considered. At the start of the outbreak, the population that responds the most to a perturbation along the direction of $a_{e,f}$ is that of the infected (IP), followed closely by that of the exposed (EP). The response of the susceptible (SP) is much smaller, being the smallest in the case of the slowest 5th wave (−0.08) and the largest in the fastest 6th wave (−0.26). As the process is driven towards the end of the explosive stage, the response of all populations intensifies. The more pronounced intensification is exhibited by the susceptible individuals (SP), which is the strongest in the case of the slowest 5th wave (from −0.08 to −2.73 the Po of SP) and the weakest in the fastest 6th wave (from −0.26 to −0.64 the Po of SP). This finding highlights the significance of the available starting population in the sequence SP→EP→IP.

The differences identified in the dynamics of the outbreak of the three epidemic waves under consideration here are consistent with the increasing influence of the vaccination that was initiated during January 2021 [85] and to the transition from the Delta variant that was the main driver during the 4th and 5th waves to the Omicron variant that was the main driver during the 6th wave [86,87,88]. For example, considering the 5th wave, the stronger opposition to $\tau_{e,f}$ from Path 4 (IP → RP) and the weaker contribution of Path 2 (EP → IP), relative to the contributions during the 4th wave, are consistent with the increased percentage of individuals in all populations being vaccinated during the 5th wave. The fact that the development of the 5th wave was less steep than the 4th wave is consistent with this influence of Paths 4 and 2. Similarly, considering the 6th wave, the increased contribution of Path 2 to $\tau_{e,f}$, relative to the contributions during the 4th and 5th waves, is consistent with the shorter incubation period and enhanced immune evasion of the Omicron variant to the general population, which resulted in the steepest outbreak development.

These conclusions were reached by focusing on the dominant explosive dynamics. Such conclusions are more robust than the resulting profiles of variables. This can be schematically demonstrated by considering the solution of the simple model dy/dt=ay; i.e., $y=y\left(0\right)e^{at}$. Considering a perturbation a→a(1+ϵ), where ϵ≪1, the relative error of the unperturbed with the perturbed solution is $e^{\epsilon at}-1$. This demonstrates that a bounded error in the dynamics can result in an exponentially large error in the profile of the solution.

## 5. Inflection Point for SP and Its Relation to the Explosive Dynamics

Since the fastest explosive mode was shown to (i) dominate the early stage of the outbreak and (ii) drive the process away from equilibrium, it is reasonable to examine the relation of the explosive dynamics to the rate by which the outbreak develops. Key point of this development is the timing of the appearance of inflection points in the profiles of the variables. Table 8 lists the inflection points of the susceptible population (SP), obtained on the basis of the two data sets for each wave. Also listed on this table are (i) the dates at which additional restriction measures (RM) were enacted by the Greek government and (ii) the dates marking the cessation of explosive dynamics $t_{exp}$, as they are indicated in Figure 4, Figure 6 and Figure 8 for the 4th, 5th and 6th waves, respectively. It is shown that the restriction measures were enacted either well after the end of the observation periods (Periods A and C) or close to the end of these periods (Periods B, D, E and F). As a result, the restriction measures had no influence on the various diagnostics generated by the fitted models. On the other hand, the dates when the measures were enacted either followed the days when explosive dynamics ceased to exist (models based on Periods A and B in the 4th wave), coincided with them (models based on Periods E and F in the 6th wave) or preceded them (models based on Periods C and D in the 5th wave). Therefore, the measures had no influence on the actual epidemic growth in the period where explosive dynamics were predicted in the 4th and 6th waves. In contrast, the restriction measures influenced the epidemic growth in the period where explosive dynamics were predicted in the 5th wave, in a manner that the model could not account for.

The results displayed in Table 8 demonstrate a very close correlation between the date when the inflection point of SP manifests, with the date $t_{exp}$, in which the character of the explosive component in the dynamics of the model changes to a dissipative one. Moreover, it is demonstrated that this prediction is robust when comparing the results obtained from the two data sets of each of the three epidemic waves. This correlation is justified by the fact that the explosive mode is the one that dominates the drive of the system away from equilibrium. The disappearance of the explosive mode marks the weakening of the outbreak development, which, according to the findings in Table 8 is manifested as an inflection point in the profile of SP that initiates the sequence SP → EP → IP.

**Figure 9:**
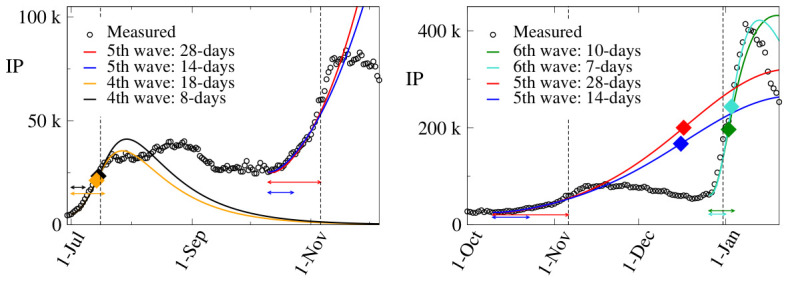
The timing of the inflection point of the SP profiles, indicated by a diamond on the IP profiles. The vertical dashed lines indicate the date on which the restriction measures were enacted.

The SP inflection point is located before the point where IP attains maximum value. This is concluded from Equation (1), which shows that in the neighborhood of $d^{2}IP/dt^{2}=0$, SP·IP=const. Since SP decreases, this implies that IP has not reached the maximum value at the SP inflection point. According to Table 8, for some cases the inflection point of SP is located beyond the fitted period (i.e., 4A, 5C, 5D), while in other cases it is located within the fitted period (4B, 6E, 6F). In any case, Figure 9 demonstrates that the peak of IP is located beyond the SP inflection point and the fitted periods. Therefore, the SP inflection point can be used as a predictive tool, although SP is not an observed variable. The robustness of this predictive relationship, with respect to parameter uncertainty, was further investigated through the practical identifiability and uncertainty quantification analyses presented in Appendix C. Although the SEIRD parameters were found to be practically non-identifiable within the available fitting windows, the quantity of interest

$$\Delta t=t_{\mathrm{peak},IP}-t_{\mathrm{inflection},SP}$$

remained positive across all sampled parameter realizations, supporting the robustness of the proposed precursor relationship.

Furthermore, as detailed in Appendix D, a forward uncertainty propagation analysis was conducted to evaluate the sensitivity of the underlying explosive time scales and stability boundaries under perturbations up to ±20%. The resulting profiles demonstrate that the parameter-induced variability envelopes of the explosive time scales remain highly contained, further confirming that the duration and terminal boundary of the explosive stage are structurally robust against parameter variations.

## 6. Discussion

A methodology for analyzing the outbreak of an epidemic wave was introduced, which focuses on revealing the mechanisms that drive or obstruct the growth of the process, instead of reproducing population profiles. This is achieved by concentrating on the dynamics that characterize the process; i.e., on the explosive mode that tends to drive the system away from equilibrium and is shown to dominate the outbreak stage. The methodology is based on the multi-scale dynamics of the process and the CSP algorithm, which allows for assessing the influence of the populations and transition paths in the model on the time scale that characterizes the action of the explosive mode. Since a fast, explosive time scale implies a strong outbreak, the contribution of the various paths to the outbreak can be assessed by the degree to which they promote or oppose the explosive character of the mode.

The new methodology was validated against the outbreak stage of three COVID-19 epidemic waves in Greece during 2021 (4th, 5th and 6th waves), using the SEIRD model. For each wave, two short observation periods were considered, starting from the same day but their length during the very early stage of the outbreak was different. This approach led to the conclusion that the methodology provides robust insights, even when based on limited early-stage data.

In all three epidemic waves considered, it was found that the outbreak growth is driven by the progression from susceptible to exposed and subsequently to infected individuals (SP → EP → IP), while recovery (IP → RP) acts as the primary opposing mechanism. However, significant differences were revealed in the influence of these paths in driving the outbreak, which were shown to reflect the differences in the growth rate of the three waves; the 6th wave exhibited the fastest growth, and the 5th wave the slowest. During the fastest 6th wave, progression EP → IP was identified as the main driving path, providing the largest contribution in all three cases considered, while the opposing influence of IP → RP was the smallest. In contrast, during the slowest 5th wave, transmission SP → EP was shown to be the main driver, while the opposing influence of IP → RP was the largest in all three waves.

These findings suggest that the intensity of the outbreak onset (i) is stronger when EP → IP dominates than when SP → EP does and (ii) is weaker as the influence of IP → RP increases. These findings were shown consistent with (i) the epidemiological transition from Delta-dominated 4th and 5th waves, which were characterized by transmission-driven growth, to the Omicron-dominated 6th wave that was characterized by progression-driven growth and (ii) the enhanced population immunity due to the Delta-based vaccination campaign during the 4th and 5th wave, which could not address the Omicron variant during the following 6th wave.

A key finding emerging from the CSP analysis was the correlation between the inflection point in the profile of the susceptible population and the disappearance of explosive dynamics, which marks the weakening of the outbreak growth away from equilibrium. This correlation was observed in all three epidemic waves and remained robust with respect to the different observation windows employed for model calibration. The results indicate that the disappearance of explosive dynamics reflects the onset of reduced effectiveness of the SP→ EP →IP cascade. Consequently, the SP inflection point was identified as a robust precursor of the IP peak. In cases where restrictive measures do not significantly interfere with the intrinsic explosive outbreak dynamics (as in the 4th and 6th waves), this inflection point can provide a reliable precursor to the peak of the infected population. In contrast, when restrictive measures are implemented during the explosive stage (5th wave), the natural evolution of the system is altered, and the predictive capability based on intrinsic dynamics is affected.

To assess the stability of this CSP-derived precursor relationship under parameter uncertainty, additional practical identifiability and uncertainty quantification analyses were performed, since the predictive usefulness of the SP inflection point depends critically on the robustness of its temporal ordering relative to the IP peak. The analysis showed that this ordering remained robust under substantial parameter perturbations across all cases considered. Specifically, the SP inflection point was consistently found to precede the IP peak despite parameter non-identifiability. Furthermore, a forward uncertainty propagation framework via Latin Hypercube Sampling was introduced to directly evaluate the sensitivity of the underlying primary CSP quantities. This analysis revealed that the parameter-induced variability envelopes of the explosive time scales remain highly contained in most cases, confirming that the duration and end date of the explosive dynamic stage are structurally robust against parameter variations.

Although the proposed framework demonstrated robust qualitative behavior across different epidemic waves and fitting windows, several important extensions remain to be explored. The present analysis was based on a relatively simple SEIRD formulation and, while it is designed to extract embedded dynamical characteristics that are not specific to a particular country or epidemic wave, was demonstrated retrospectively using COVID-19 epidemic data from Greece. Future studies should therefore investigate the predictive capability of the framework through out-of-sample validation, application to additional epidemic settings, and comparison against alternative epidemic forecasting and early-warning methodologies. Moreover, extensions incorporating additional epidemiological mechanisms, such as vaccination dynamics and reinfection effects, could further clarify the robustness and generalizability of the proposed multiscale framework. Such extensions would further clarify the robustness, generalizability, and predictive capabilities of the proposed multiscale framework.

The proposed CSP-based time scale framework provides a robust, dynamically grounded and interpretable approach for analyzing epidemic outbreaks, focusing on the intrinsic dynamics of the governing equations rather than on solution profiles. By identifying the dominant explosive mode and its associated mechanisms, the methodology enables the extraction of meaningful dynamical information from limited early-stage data, demonstrating robustness across different observation windows. The results show that the framework can consistently capture the key processes driving outbreak initiation and attenuation, while providing mechanistic insight into the roles of transmission, progression, and recovery. In this context, the analysis establishes a direct link between dynamical behavior and observable epidemic features, offering a principled basis for early outbreak assessment.

Crucially, the identification of the susceptible inflection point does not require direct real-time measurement of the susceptible population. Instead, the proposed framework estimates the timing of this transition indirectly through CSP-based analysis of limited early-stage epidemic data. In this sense, the methodology may provide a useful computational indicator for assessing changes in outbreak growth dynamics and the onset of outbreak attenuation. Consequently, the framework could potentially support epidemic monitoring and early-stage decision-making, particularly in settings where limited observational data are available. More generally, the results suggest that time-scale-based CSP analysis may offer a promising and interpretable approach for real-time epidemic assessment and the investigation of intrinsic outbreak dynamics.

## Figures and Tables

**Table 1:** Translational Glossary: Mapping CSP terminology to biological and epidemiological interpretation.

Mathematical Term (CSP)	Biological/Epidemiological Translation	Practical Significance in This Study
Mode ($a_{n}f^{n}$)	Dynamical component	A component of the model, the action of which is characterized by an explosive or dissipative time scale. The mode that is characterized by the fastest time scale drives the initial surge, while all other modes sustain or attenuate the epidemic wave before stabilization occurs.
Explosive time scale ($\tau_{e}$)	Characteristic expansion time	Represents the time frame of the action of a mode (explosive) that tends to drive the system away from equilibrium. A smaller value (e.g., ∼4 days during the 6th wave) indicates a more aggressive and rapidly spreading variant compared to larger values (e.g., ∼8 days during the 4th wave or ∼28 days during the 5th wave).
Dissipative time scale ($\tau_{d}$)	Stabilization or damping rate	Represents the time frame of the action of a mode (dissipative) that tends to drive the system towards equilibrium. When a dissipative mode dominates, the outbreak transitions into decay and active cases begin to decline.
Slow Invariant Manifold (SIM)	Epidemic trajectory	The reduced-dimensional path followed by the epidemic once fast transient processes subside. It represents the established dynamical regime during the outbreak phase.
Amplitude (*f*)	Driver intensity	Provides a measure of the impact of a mode in driving the epidemic wave. In the case of an explosive mode, a high amplitude indicates dominant transmission dynamics, while a low amplitude signals increasing control or immunity effects.
Time Scale Participation Index (TPI)	Mechanism identifier	Quantifies the contribution of individual biological transitions (e.g., transmission, incubation, recovery) to the time scale. In the case of an explosive time scale, it determines the degree to which the outbreak is promoted by high transmission ($R^{1}$) or by rapid progression from exposure to infection ($R^{2}$), or it is obstructed by recovery ($R^{4}$).
Pointer (Po)	Population influence index	Identifies which population compartment most strongly influenced by a specific mode. For example, a high Pointer value for the exposed population during the 6th wave indicates that rapid progression from exposure to infection drives the surge.
Inflection point	Turning point in epidemic acceleration	The moment when outbreak growth shifts from acceleration to deceleration. This precedes the peak of infected population and serves as an early warning indicator of the impending peak.

**Table 2:** The parameters β, σ, μ and γ, as were estimated for the SEIRD model at each period of the 4th, 5th and 6th waves.

	Wave 4		Wave 5		Wave 6	
	Period A	Period B	Period C	Period D	Period E	Period F
	1/7/21–8/7/21	1/7/21–18/7/21	10/10/21–23/10/21	10/10/21–06/11/21	26/12/21–1/1/22	26/12/21–4/1/22
β	6.367 × 10^−6^	8.336 × 10^−6^	6.588 × 10^−8^	6.964 × 10^−8^	1.995 × 10^−6^	3.049 × 10^−6^
σ	1.096 × 10^−1^	1.110 × 10^-1^	7.190 × 10^−2^	6.410 × 10^−2^	7.627 × 10^−2^	4.643 × 10^−2^
μ	9.131 × 10^−4^	6.113 × 10^−4^	1.156 × 10^−3^	1.133 × 10^−3^	6.322 × 10^−4^	4.785 × 10^−4^
γ	3.777 × 10^−2^	3.894 × 10^−2^	6.735 × 10^−2^	6.306 × 10^−2^	7.522 × 10^−2^	4.643 × 10^−2^

**Table 3:** Fourth wave. The major contributions to $\tau_{e,f}$ from the paths in the model (largest TPIs, $J_{k}^{e,f}$) and the populations related the most to the fastest explosive mode (largest Pos, $D_{n}^{e,f}$), computed at the start of the 4th wave (July 1) and near the end of the explosive stage (July 7). $R^{1}:\;SP\to EP$, $R^{2}:\;EP\to IP$, $R^{3}:\;IP\to DP$ and $R^{4}:\;IP\to RP$.

Period A						Period B					
$J_{k}^{e,f}$			$D_{n}^{e,f}$			$J_{k}^{e,f}$			$D_{n}^{e,f}$		
	Jul-1	Jul-7		Jul-1	Jul-7		Jul-1	Jul-7		Jul-1	Jul-7
$R^{1}$	0.51	0.44	*IP*	0.69	0.99	*R* ^1^	0.50	0.38	*IP*	0.71	1.34
$R^{2}$	0.35	0.31	EP	0.49	0.62	$R^{2}$	0.36	0.31	EP	0.51	0.80
$R^{4}$	−0.14	−0.24	SP	−0.18	−0.61	$R^{4}$	−0.14	−0.30	SP	−0.22	−1.14
$R^{3}$	0.00	−0.01				$R^{3}$	0.00	−0.01			

**Table 4:** Fifth wave. The major contributions to $\tau_{e,f}$ from the paths in the model (largest TPIs, $J_{k}^{e,f}$) and the populations related the most to the fastest explosive mode (largest Pos, $D_{n}^{e,f}$), computed at the start of the 5th wave (October 10) and near the end of the explosive stage (November 10). $R^{1}:\;SP\to EP$, $R^{2}:\;EP\to IP$, $R^{3}:\;IP\to DP$ and $R^{4}:\;IP\to RP$.

Period C						Period D					
$J_{k}^{e,f}$			$D_{n}^{e,f}$			$J_{k}^{e,f}$			$D_{n}^{e,f}$		
	Oct-10	Nov-20		Oct-10	Nov-20		Oct-10	Nov-20		Oct-10	Nov-20
$R^{1}$	0.49	0.43	IP	0.55	1.90	$R^{1}$	0.49	0.43	EP	0.54	0.92
$R^{4}$	−0.34	−0.46	EP	0.53	1.83	$R^{4}$	−0.31	−0.42	IP	0.53	0.92
$R^{2}$	0.16	0.10	SP	−0.08	−2.73	$R^{2}$	0.19	0.14	SP	−0.07	−0.84
$R^{3}$	−0.01	−0.01				$R^{3}$	−0.01	−0.01			

**Table 5:** Sixth wave. The major contributions to $\tau_{e,f}$ from the paths in the model (largest TPIs, $J_{k}^{e,f}$) and the populations related the most to the fastest explosive mode (largest Pos, $D_{n}^{e,f}$), computed at the start of the 6th wave (December 26) and near the end of the explosive stage (December 29). $R^{1}:\;SP\to EP$, $R^{2}:\;EP\to IP$, $R^{3}:\;IP\to DP$ and $R^{4}:\;IP\to RP$.

Period E						Period F					
$J_{k}^{e,f}$			$D_{n}^{e,f}$			$J_{k}^{e,f}$			$D_{n}^{e,f}$		
	Dec-26	Dec-29		Dec-26	Dec-29		Dec-26	Dec-29		Dec-26	Dec-29
$R^{2}$	0.45	0.46	IP	0.63	0.82	$R^{2}$	0.53	0.55	IP	0.67	1.03
$R^{1}$	0.41	0.32	EP	0.63	0.82	$R^{1}$	0.37	0.22	EP	0.68	1.03
$R^{4}$	−0.13	−0.22	SP	−0.26	−0.64	$R^{4}$	−0.10	−0.23	SP	−0.33	−1.06
$R^{3}$	−0.01	0.00				$R^{3}$	0.00	0.00			

**Table 6:** Comparison of the characteristic time scale $\tau_{e,f}$ during the 4th, 5th and 6th waves. $\tau_{e,f}^{0}$ and $\tau_{e,f}^{*}$ represent the value of the time scale at the start of each wave and at the end of the corresponding explosive stage, respectively; see Table 3, Table 4 and Table 5 for the dates accounted for. The value of the fast dissipative time scale at the end of the explosive stage $\tau_{d}^{*}$ is also listed.

Wave 4		Wave 5		Wave 6	
Period A	Period B	Period C	Period D	Period E	Period F
$\tau_{e,f}^{0}$ = 7.5 d	$\tau_{e,f}^{0}$ = 7.2 d	$\tau_{e,f}^{0}$ = 29.9 d	$\tau_{e,f}^{0}$ = 26.7 d	$\tau_{e,f}^{0}$ = 3.8 d	$\tau_{e,f}^{0}$ = 3.9 d
$\tau_{e,f}^{*}$ = 28.9 d	$\tau_{e,f}^{*}$ = 28.2 d	$\tau_{e,f}^{*}$ = 78.8 d	$\tau_{e,f}^{*}$ = 74.4 d	$\tau_{e,f}^{*}$ = 19.0 d	$\tau_{e,f}^{*}$ = 26.5 d
$\tau_{d}^{*}$ = 3.4 d	$\tau_{d}^{*}$ = 3.3 d	$\tau_{d}^{*}$ = 6.1 d	$\tau_{d}^{*}$ = 6.4 d	$\tau_{d}^{*}$ = 2.0 d	$\tau_{d}^{*}$ = 2.0 d

**Table 7:** The range of major contributions of the paths to $\tau_{e,f}$ (as measured by the TPI indices) and the range of association of the variables to the mode $a_{e,f}f^{e,f}$ (as measured by the Po indices), during the explosive stage of the 4th, 5th and 6th waves; data from the start and the end of the explosive stage of Periods A, C and E, respectively, as stated in Table 3, Table 4 and Table 5.

Wave 4 (Period A)		Wave 5 (Period C)		Wave 6 (Period E)	
$R^{1}$:	0.51 − 0.44	$R^{1}$:	0.49 − 0.43	$R^{2}$:	0.45 − 0.46
$R^{2}$:	0.35 − 0.31	$R^{4}$:	−0.34 − −0.46	$R^{1}$:	0.41 − 0.32
$R^{4}$:	−0.14 − −0.24	$R^{2}$:	0.16 − 0.10	$R^{4}$:	−0.13 − −0.22
$R^{3}$:	−0.00 − −0.01	$R^{3}$:	−0.01 − −0.01	$R^{3}$:	−0.01 − −0.00
IP:	0.69 − 0.99	IP:	0.55 − 1.90	IP:	0.63 − 0.82
EP:	0.49 − 0.62	EP:	0.53 − 1.83	EP:	0.63 − 0.82
SP:	−0.18 − −0.61	SP:	−0.08 − −2.73	SP:	−0.26 − −0.64

**Table 8:** Inflection point in the profile of the susceptible population (SP) during the three epidemic waves, on the basis of the two data sets for each wave (Periods A–F). Also listed are: (i) the date range for the data sets employed for model fitting, (ii) the dates when additional restriction measures (RM) were implemented by the Greek government, and (iii) the date marking the cessation of explosive dynamics $t_{exp}$, as shown in Figure 4, Figure 6 and Figure 8 for the 4th, 5th and 6th waves, respectively.

Wave	Period		RM	$t_{\exp}$	*SP* Inflection Points
4	A	(Jul-1 to Jul-8)	Jul-16	Jul-14	Jul-12
	B	(Jul-1 to Jul-18)	Jul-16	Jul-12	Jul-10
5	C	(Oct-10 to Oct-23)	Nov-6	Jan-2	Jan-1
	D	(Oct-10 to Nov-6)	Nov-6	Dec-30	Dec-28
6	E	(Dec-26 to Jan-1)	Dec-30	Jan-1	Dec-31
	F	(Dec-26 to Jan-4)	Dec-30	Dec-31	Dec-30

**Figure A1:**
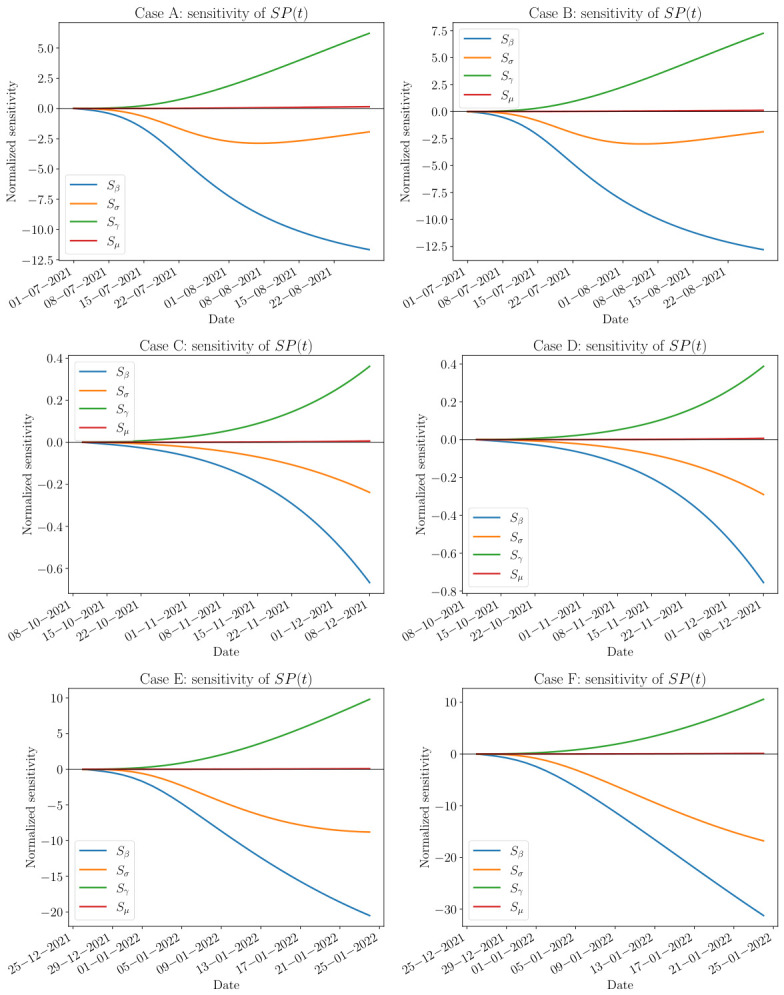
Normalized local sensitivity profiles of the susceptible population SP(t) with respect to the SEIRD parameters β, σ, γ, and μ for Cases A–F. The profiles indicate that β, σ, and γ are the dominant parameters influencing the susceptible population across all fitting windows.

**Figure A2:**
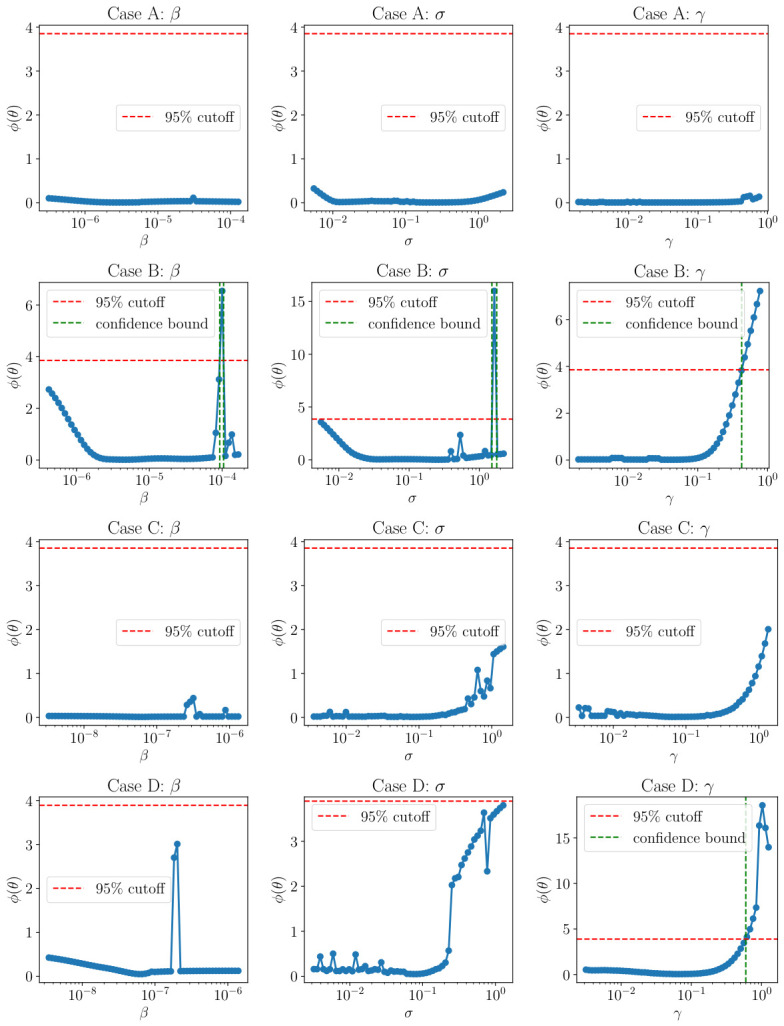

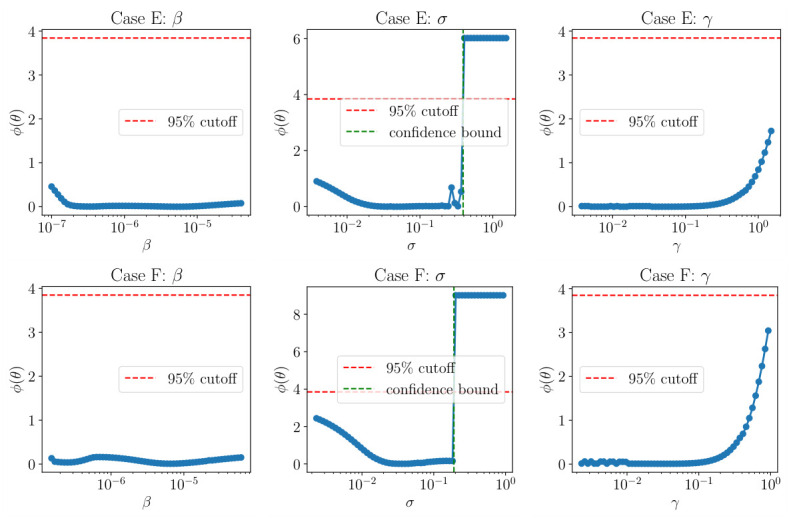
One-dimensional profile likelihoods for the parameters β, σ, and γ across all cases. Each row corresponds to a case (A–F), and each column corresponds to a parameter. The dashed horizontal line indicates the 95% likelihood threshold. The absence of intersections with this threshold within the explored parameter ranges indicates lack of practical identifiability.

**Figure A3:**
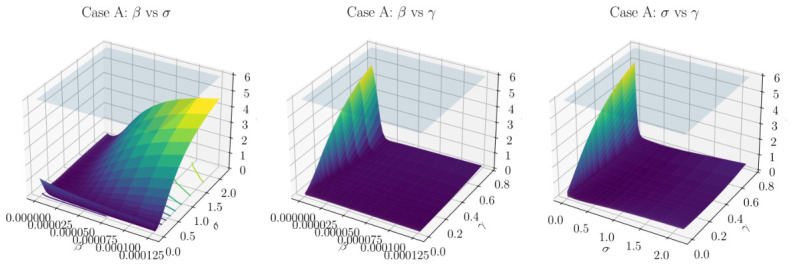

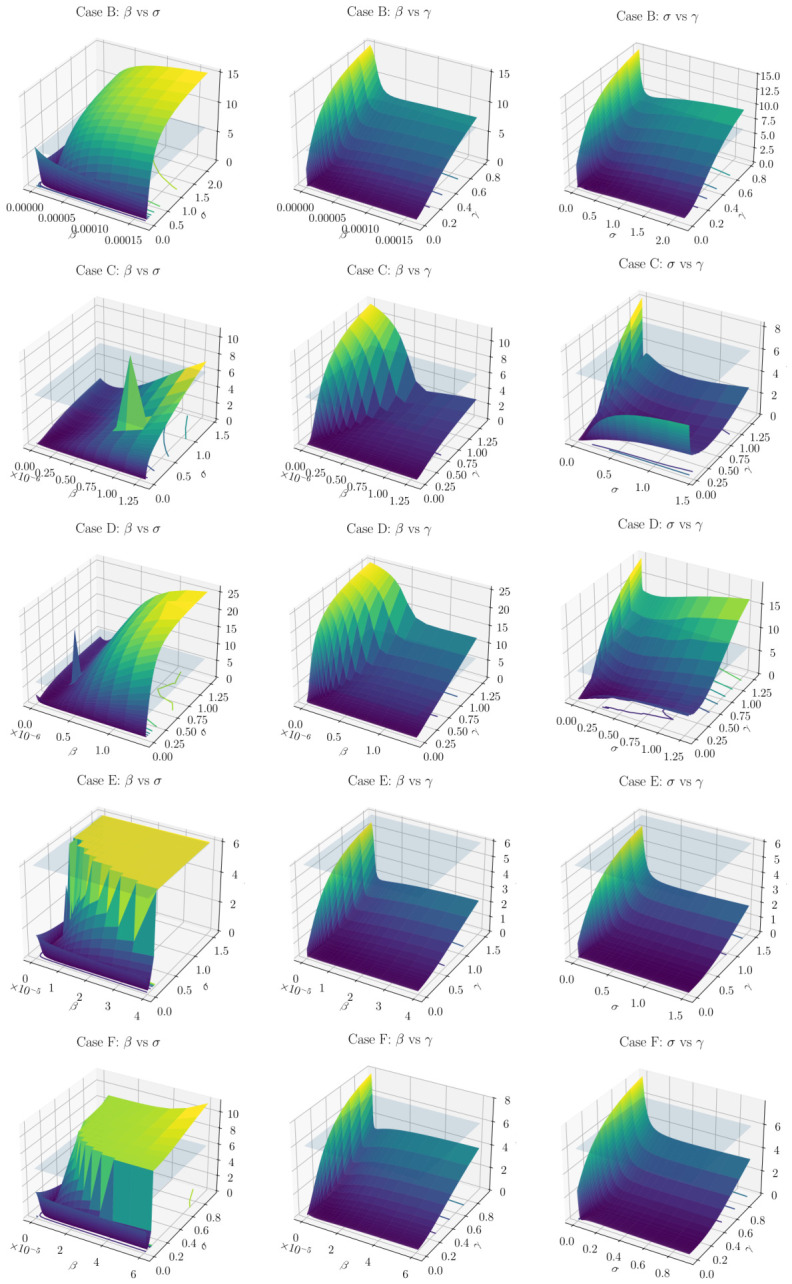
Two-dimensional profile likelihood surfaces for parameter pairs (β,σ), (β,γ), and (σ,γ) across all cases. Each row corresponds to a case (A–F), and each column corresponds to a parameter pair. The elongated valleys and flat regions indicate strong parameter correlations and compensation effects, confirming the lack of practical identifiability.

**Figure A4:**
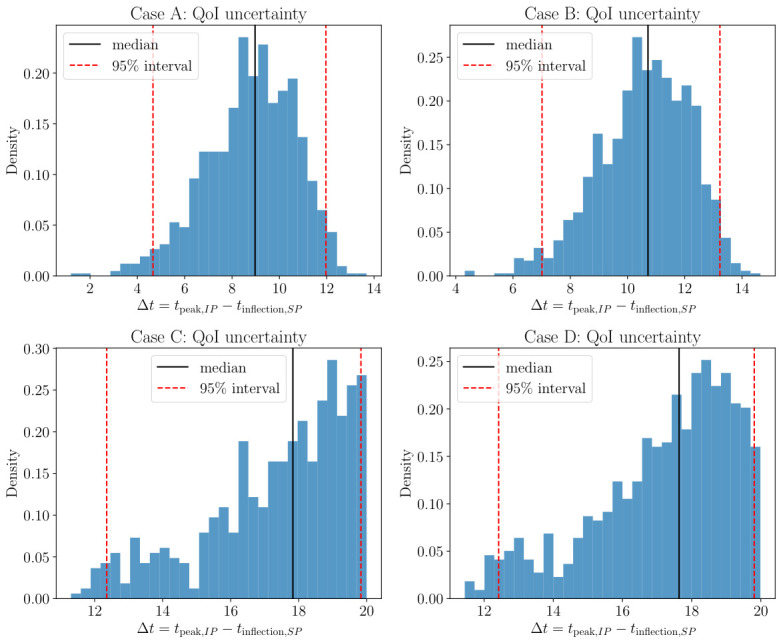

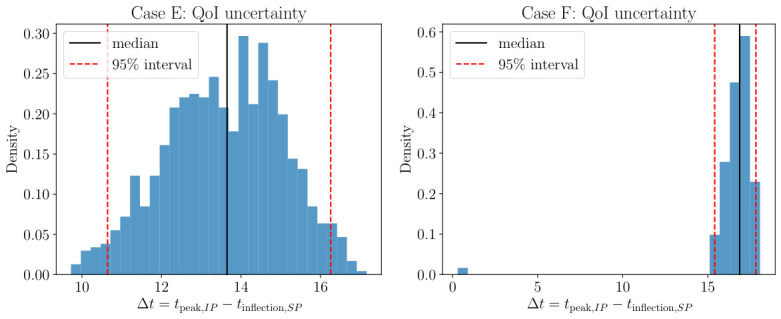
Monte Carlo uncertainty quantification of the quantity of interest $\Delta t=t_{\mathrm{peak},I}-t_{\mathrm{inflection},S}$ for Cases A–F. The histograms show the density of Δt obtained from parameter perturbations of 20% around the calibrated values. In all cases, the distribution remains strictly positive, indicating that the susceptible-population inflection point precedes the infected-population peak.

**Figure A5:**
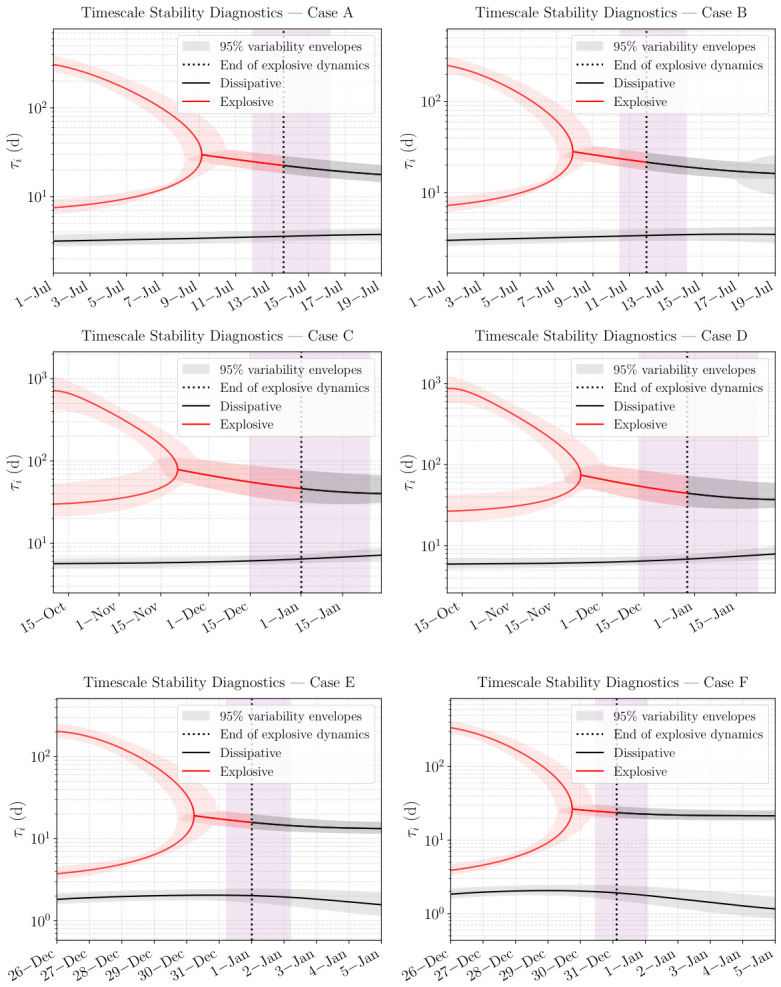
Evolution of analytical dynamical time scales accompanied by parameter-induced variability envelopes across all calibrated epidemic waves (Cases A–F). Shaded regions denote the forward propagation of a uniform ±20% parameter perturbation box evaluated via Latin Hypercube Sampling. Solid lines refer to the nominal baseline system, intersected by the reference critical transition date (vertical dotted line) and its corresponding temporal uncertainty band.

**Figure A6:**
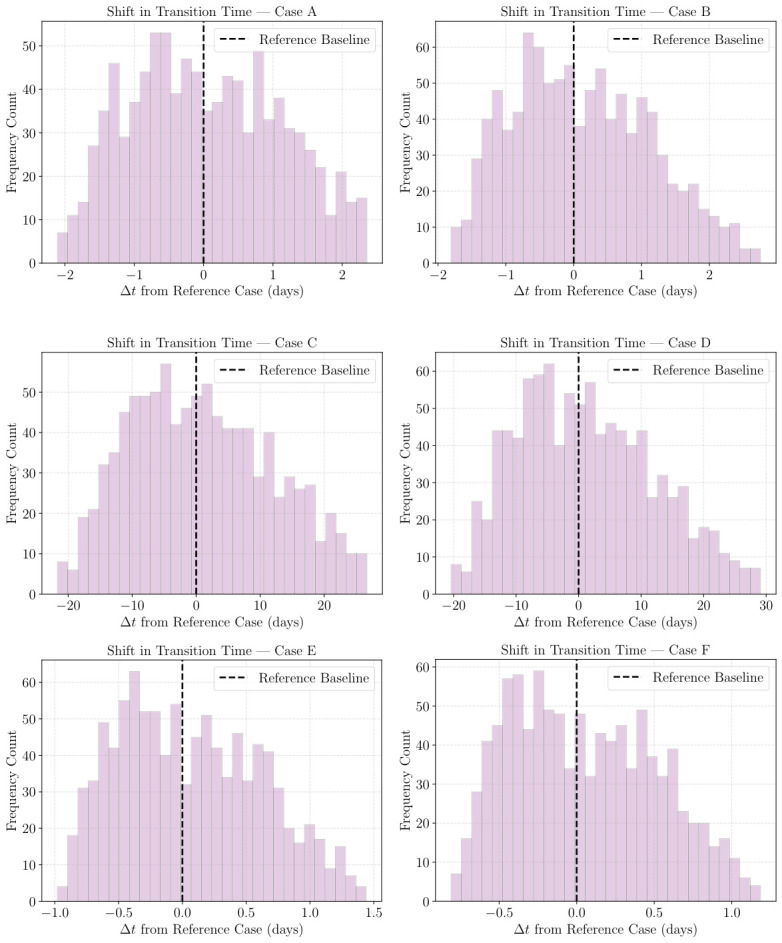
Statistical frequency distributions of the critical transition dates ($\Delta t_{\mathrm{crit}}$) for Cases A–F under a ±20% parameter-induced uncertainty design. All individual panels illustrate the deviation from the unperturbed system and are centered symmetrically around the nominal reference calibration baseline (0), marked via a primary dashed indicator line.

**Figure A7:**
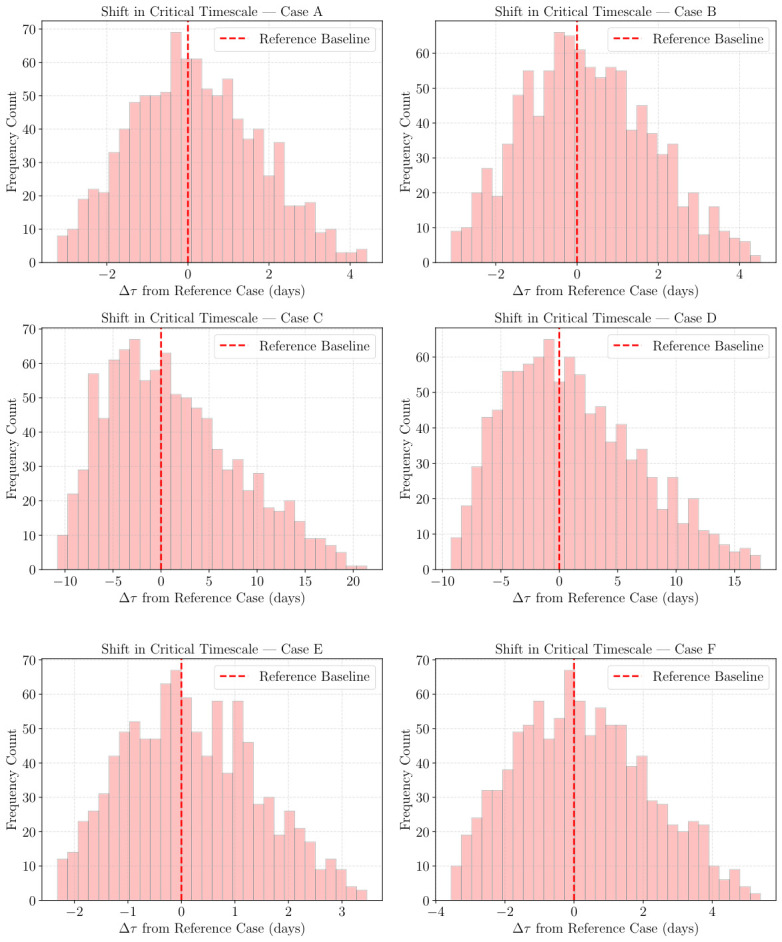
Statistical frequency distributions of the critical overlapping complex timescale values ($\Delta \tau_{\mathrm{crit}}$) for Cases A–F under a ±20% parameter-induced uncertainty design. All individual panels illustrate the variation relative to the unperturbed system and are centered symmetrically around the nominal reference calibration baseline (0), marked via a primary dashed indicator line.

## Data Availability

All data reported in this manuscript are available from the corresponding author upon request.

## Abbreviations

The following abbreviations are used in this manuscript:

CSP	Computational Singular Perturbation method
TPI	Time scale Participation Index
Po	CSP Pointer
API	Amplitude Participation Index

## Appendix A. COPASI Calibration Setup

This appendix summarizes the parameter-estimation setup employed in the COPASI-based calibration of the SEIRD model for all epidemic waves considered in the present study. The practical identifiability and uncertainty quantification analyses associated with these calibrations are presented separately in Appendix C.

### Appendix A.1. Parameter Estimation Framework

The epidemic-wave calibrations were performed using the parameter-estimation framework implemented in COPASI (Version 4.34, Build 251). The optimization procedure employed the Genetic Algorithm SR method to minimize a weighted least-squares objective function of the form

$$\phi \left(\theta \right)=\sum_{k}w_{k}{\left(y_{\mathrm{model}}\left(t_{k};\theta \right)-y_{\mathrm{data}}\left(t_{k}\right)\right)}^{2},$$

where $y_{\mathrm{model}}$ and $y_{\mathrm{data}}$ denote the model predictions and the reported epidemic data, respectively. The calibration was performed using the reported active infected, recovered, and deceased populations. Depending on the epidemic wave and fitting period, the fitting windows varied between 7 and 28 days.

### Appendix A.2. Optimization Settings

For all epidemic-wave calibrations, the COPASI optimization procedure employed the Genetic Algorithm SR method using 10,000 generations, a population size of 20, mutation variance equal to 0.1, Pf parameter equal to 0.475, and random seed equal to 0.

### Appendix A.3. Model Parameters and Bounds

The calibrated SEIRD parameters were

β,σ,γ,μ.

Within the COPASI implementation, these parameters were represented through the quantities

BINSP,aincp,aip,muINSP,

according to the relations

$$\beta =\frac{\mathrm{BINSP}}{c},\;\sigma =\frac{1}{\mathrm{aincp}},\;\gamma =\frac{1}{\mathrm{aip}},\;\mu =\mathrm{muINSP}.$$

The initial susceptible and exposed populations were also estimated during calibration.

Table A1 summarizes the parameter bounds and fitted quantities for all epidemic waves.

**Table A1:** COPASI parameter bounds and fitted values for all epidemic-wave calibrations.

Case	Parameter	Lower Bound	Upper Bound	Fitted Value
A	aincp	1	16.5	9.1206
A	aip	0.001	27	26.4747
A	BINSP	$10^{-4}$	10	0.473678
A	muINSP	$10^{-4}$	1	$9.1308\times 10^{-4}$
A	c	1000	75,000	74,394.3498
A	EP_est	1000	∞	3430.6532
B	aincp	1	16.5	9.0071
B	aip	0.001	27	25.6804
B	BINSP	$10^{-4}$	10	0.523070
B	muINSP	$10^{-4}$	1	$6.1132\times 10^{-4}$
B	c	1000	75,000	62,746.2000
B	EP_est	1000	∞	2844.2800
C	aincp	1	16.5	13.9082
C	aip	0.001	27	14.8471
C	BINSP	$10^{-4}$	10	0.156658
C	muINSP	$10^{-4}$	1	$1.1563\times 10^{-3}$
C	c	1000	$5\times 10^{6}$	2,378,020
C	EP_est	1000	∞	23,043
D	aincp	1	16.5	15.6006
D	aip	0.001	27	15.8587
D	BINSP	$10^{-4}$	10	0.168556
D	muINSP	$10^{-4}$	1	$1.1329\times 10^{-3}$
D	c	1000	$5\times 10^{6}$	2,420,650
D	EP_est	1000	∞	20,653
E	aincp	1	30	13.1102
E	aip	0.001	30	13.2942
E	BINSP	$10^{-4}$	10	2.25715
E	muINSP	$10^{-4}$	1	$6.3221\times 10^{-4}$
E	c	1000	$5\times 10^{6}$	1,131,470
E	EP_est	0	∞	0.0230475
F	aincp	1	30	21.5378
F	aip	0.001	30	21.5378
F	BINSP	$10^{-4}$	10	3.45622
F	muINSP	$10^{-4}$	1	$4.7851\times 10^{-4}$
F	c	1000	$5\times 10^{6}$	1,133,680
F	EP_est	0	∞	0.0826524

Following the parameter calibration, the accuracy of the resulting model fits was evaluated. Table A2 presents the computed Mean Squared Error (MSE) and Root Mean Squared Error (RMSE) for the overall multi-variable fit as well as individual evaluations for the tracking trajectories of the infected (IP), recovered (RP), and deceased (DP) populations across all studied cases.

**Table A2:** Model Fitting Performance Metrics (MSE and RMSE) across Cases A–F.

	Overall		IP		RP		DP	
Case	MSE	RMSE	MSE	RMSE	MSE	RMSE	MSE	RMSE
A	$3.85\times 10^{5}$	620.86	$9.88\times 10^{5}$	994.10	$1.68\times 10^{5}$	409.98	81.59	9.03
B	$8.10\times 10^{5}$	900.00	$2.27\times 10^{6}$	1506.56	$1.60\times 10^{5}$	399.94	313.73	17.71
C	$2.07\times 10^{6}$	1438.39	$1.06\times 10^{6}$	1028.38	$5.15\times 10^{6}$	2269.01	912.71	30.21
D	$1.11\times 10^{7}$	3328.74	$5.15\times 10^{6}$	2269.86	$2.81\times 10^{7}$	5299.70	2489.93	49.90
E	$2.13\times 10^{8}$	14,588.81	$5.66\times 10^{8}$	23,788.11	$7.26\times 10^{7}$	8521.83	3898.38	62.44
F	$2.18\times 10^{8}$	14,776.80	$6.09\times 10^{8}$	24,682.44	$4.58\times 10^{7}$	6769.72	9973.49	99.87

### Appendix A.4. Goodness of Fit and Robustness

The quality of the calibrations was assessed based on the agreement between the simulated epidemic trajectories and the reported epidemic data within the fitting windows. The resulting model trajectories exhibited close agreement with the observed active infected, recovered, and deceased populations across all epidemic waves.

Additional practical identifiability and uncertainty quantification analyses were performed independently in Python v3.11.9 in order to evaluate the robustness of the calibrated parameters and the CSP-derived dynamical conclusions. These analyses included sensitivity analysis, one- and two-dimensional profile likelihood analysis, and Monte Carlo uncertainty quantification, and are presented in Appendix C.

## Appendix B. The Origin of the Fast Dissipative Mode

The fastest mode $a_{d}f^{d}$ tends to drive the system to equilibrium, since the time scale $\tau_{d}$ that characterizes its action is dissipative. Listed in Table A3 are indicative TPI indices, $J_{k}^{d}$, that measure the relative contribution of the various paths in the model to the time scale $\tau_{d}$. These values were computed right before the end of the explosive stage; 7 July 2021 for Periods A and B, 20 November 2021 for Periods C and D, and 29 December 2021 for Periods E and F. It is shown that in all three waves considered, all four paths contribute to the dissipative character of the mode ($J_{k}^{d}<0$); Paths 1 and 2 exhibiting consistently the largest contributions and Path 3 exhibiting the smallest. Since these are the paths in the sequence SP → EP → IP → RP/DP, it is concluded that this mode tends to bring the process to completion.

As stated in Section 2.5 and shown in Figure 4, Figure 6 and Figure 8, this dissipative mode is not the dominant one during the outbreak stage because the magnitude of its amplitude $f^{d}$:

(A1) $$f^{d}=\left(b^{d}\cdot S_{1}\right)R^{1}+\left(b^{d}\cdot S_{2}\right)R^{2}+\left(b^{d}\cdot S_{3}\right)R^{3}+\left(b^{d}\cdot S_{4}\right)R^{4}$$

remains small, due to significant cancellations among the additive terms; i.e., $f^{d}/max\left|\left(b^{d}\cdot S_{k}\right)R^{k}\right|\ll 1$. A measure of these cancellations is obtained by the Amplitude Participation Index (API) [95,96], defined as:

(A2) $$P_{k}^{d}=\frac{p_{k}^{d}}{\sum_{i=1}^{4}\left|p_{i}^{d}\right|},\;\;p_{k}^{n}=\left(b^{d}\cdot S_{k}\right)R^{k},\;\;|P_{1}^{d}\left|+\ldots \right|P_{4}^{d}|=1.$$

The major values of $P_{k}^{d}$ that are listed on Table A3 for all three waves considered and show that significant cancellations occur between the sum of terms relating to Paths 1, 3 and 4 with the term relating to Path 2; the major cancellations recorded between Paths 1 and 2.

The features displayed in Table A3 are in qualitative agreement with the leading order expressions for the CSP basis vectors and time scale related to the fastest mode $a_{d}f^{d}$ that are obtained with the CSP-based refinement algorithm [68,69]:

(A3) $$\tau_{d}={\left(\beta IP+\sigma \frac{\beta SP+\sigma }{\beta IP+\sigma }\right)}^{-1},$$

(A4) $$a_{d}={\left[\begin{array}{ccccc} -\frac{\beta IP}{\beta IP+\sigma } & 1 & -\frac{\sigma }{\beta IP+\sigma } & 0 & 0 \end{array}\right]}^{⊤},$$

(A5) $$b^{d}={\left[\begin{array}{ccccc} -\beta IP\tau_{d} & \sigma \tau_{d} & -\beta SP\tau_{d} & 0 & 0 \end{array}\right]}^{⊤}.$$

The expressions in Equations (A3) and (A4) show that $\tau_{d}$ is mainly generated by Paths 1 and 2 and that the $a_{d}f^{d}$ mode tends to deplete SP and IP and restore EP, respectively. The expression in (A5) leads to the following expression of $f^{d}$:

(A6) $$f^{d}=R^{1}\left(\beta IP+\sigma \right)\tau_{d}-R^{2}\left(\beta SP+\sigma \right)\tau_{d}+R^{3}\left(\beta SP\right)\tau_{d}+R^{4}\left(\beta SP\right)\tau_{d},$$

which is in qualitative agreement with the results displayed in Table A3.

**Table A3:** Percentage major contributions of the various paths to (i) the fast dissipative time scale $\tau_{d}$ (as measured by the TPI, $J_{k}^{d}$) (top) and (ii) the related amplitude $f^{b}$ (as measured by the API, $P_{k}^{d}$) (bottom), estimated right before the end of the explosive stage; 7 July 2021 for Periods A and B, 20 November 2021 for Periods C and D, and 29 December 2021 for Periods E and F.

Wave 4-Jul. 7		Wave 5-Nov. 20		Wave 6-Dec. 29	
Period A	Period B	Period C	Period D	Period E	Period F
** $J_{k}^{d}$ **					
$R^{2}$: −0.53	$R^{2}$: −0.51	$R^{2}$: −0.50	$R^{2}$: −0.49	$R^{1}$: −0.53	$R^{1}$: −0.65
$R^{1}$: −0.42	$R^{1}$: −0.44	$R^{1}$: −0.30	$R^{1}$: −0.31	$R^{2}$: −0.41	$R^{2}$: −0.32
$R^{4}$: −0.05	$R^{4}$: −0.05	$R^{4}$: −0.19	$R^{4}$: −0.19	$R^{4}$: −0.06	$R^{4}$: −0.03
$R^{3}$: −0.00	$R^{3}$: −0.00	$R^{3}$: −0.01	$R^{3}$: −0.01	$R^{3}$: −0.00	$R^{3}$: −0.00
** $P_{k}^{d}$ **					
$R^{2}$: −0.47	$R^{1}$: 0.47	$R^{2}$: −0.50	$R^{2}$: −0.50	$R^{1}$: 0.49	$R^{1}$: 0.57
$R^{1}$: 0.46	$R^{2}$: −0.46	$R^{1}$: 0.30	$R^{1}$: 0.31	$R^{2}$: −0.42	$R^{2}$: −0.37
$R^{4}$: 0.07	$R^{4}$: 0.07	$R^{4}$: 0.20	$R^{4}$: 0.19	$R^{4}$: 0.09	$R^{4}$: 0.06
$R^{3}$: 0.00	$R^{3}$: 0.00	$R^{3}$: 0.00	$R^{3}$: 0.00	$R^{3}$: 0.00	$R^{3}$: 0.00

## Appendix C. Identifiability and Robustness Analysis

In order to assess the reliability of the calibrated SEIRD model and the robustness of the derived results, we performed a systematic analysis consisting of: (i) local sensitivity analysis, (ii) profile likelihood-based identifiability assessment, and (iii) uncertainty quantification of a derived quantity of interest (QoI). This multi-step approach allows us to evaluate both the influence of model parameters on the system dynamics and the stability of the conclusions under parameter uncertainty.

### Appendix C.1. Sensitivity Analysis

For each case, we computed the local (normalized) sensitivities of the model states with respect to the parameters. For a state variable $x_{i}\left(t\right)$ and parameter $p_{j}$, the sensitivity index is defined as

$$S_{x_{i},p_{j}}\left(t\right)=\frac{p_{j}}{x_{i}\left(t\right)}\frac{\partial x_{i}\left(t\right)}{\partial p_{j}}.$$

The sensitivities were approximated numerically using central finite differences around the calibrated parameter values. This normalization allows for direct comparison of the relative importance of parameters across different variables and scales.

The analysis was performed for all state variables, with particular emphasis on the susceptible population S(t), which plays a key role in the transmission dynamics. The normalized sensitivity profiles of S(t) with respect to all model parameters are shown in Figure A1. Across all cases, the results consistently indicate that the transmission-related parameters β and σ, together with the recovery parameter γ, exert the dominant influence on S(t). These parameters were therefore selected for further identifiability analysis.

### Appendix C.2. Profile Likelihood Analysis

To assess practical identifiability, we employed the profile likelihood approach. For each parameter $p_{j}$, its value was fixed over a prescribed range, while the remaining parameters were re-optimized to minimize a weighted least-squares objective function of the form

$$\phi \left(\theta \right)=\sum_{k}w_{k}{\left(y_{\mathrm{model}}\left(t_{k};\theta \right)-y_{\mathrm{data}}\left(t_{k}\right)\right)}^{2}.$$

The explored parameter range was defined multiplicatively around the calibrated value $\theta^{*}$ as

$$\theta \in [\mathrm{scale}\text{\_}\mathrm{low}\cdot \theta^{*},\;\mathrm{scale}\text{\_}\mathrm{high}\cdot \theta^{*}],$$

subject to admissible bounds. Wide ranges were considered in order to detect potential flat regions in the likelihood landscape.

Confidence intervals were determined using the likelihood-based threshold

$$\phi \left(\theta \right)\leq \phi_{\min}+\chi_{0.95,1}^{2}.$$

#### Appendix C.2.1. One-Dimensional Profile Likelihoods

For each case, we computed the one-dimensional profile likelihoods for the dominant parameters identified by the sensitivity analysis, namely β, σ, and γ. The resulting curves are shown in Figure A2.

Across all cases, the profile likelihoods exhibit extended flat regions over several orders of magnitude in the parameter space and do not intersect the confidence threshold within the explored domain. This behavior indicates that the parameters are not practically identifiable, as large variations in their values can be compensated by re-adjustments of the remaining parameters while preserving the quality of fit.

#### Appendix C.2.2. Two-Dimensional Profile Likelihood Surfaces

To further investigate parameter dependencies and compensation effects, we computed two-dimensional profile likelihood surfaces for pairs of dominant parameters. Specifically, we considered the pairs (β,σ), (β,γ), and (σ,γ), as these parameters were identified as the most influential through the sensitivity analysis.

For each pair, the two parameters were fixed over a two-dimensional grid, while the remaining parameters were re-optimized. The resulting surfaces are shown in Figure A3.

The surfaces reveal extended flat regions and elongated valleys, indicating strong parameter correlations and compensatory effects. This confirms that different parameter combinations can produce statistically equivalent fits, further supporting the lack of practical identifiability observed in the one-dimensional analysis.

### Appendix C.3. Uncertainty Quantification of the Quantity of Interest

Given the lack of practical identifiability of the individual parameters, we next evaluated the robustness of a derived quantity of interest (QoI). The QoI was defined as

$$\Delta t=t_{\mathrm{peak},I}-t_{\mathrm{inflection},S},$$

where $t_{\mathrm{peak},I}$ denotes the time at which the infected population reaches its maximum, and $t_{\mathrm{inflection},S}$ denotes the inflection point of the susceptible population.

Since the profile likelihood analysis did not yield finite confidence intervals for the parameters, uncertainty quantification could not be performed by sampling from parameter confidence bounds. Instead, we adopted a Monte Carlo perturbation approach. Parameter sets were generated by applying multiplicative perturbations around the calibrated values. Robustness was examined for perturbation levels up to 20%. For each sampled parameter set, the SEIRD model was simulated and the corresponding value of Δt was computed.

The resulting distributions of Δt are shown in Figure A4. In all cases, the computed values satisfy

Δt>0.

Thus, the inflection point of the susceptible population consistently precedes the peak of the infected population, even under substantial parameter perturbations. This supports the robustness of the proposed temporal ordering and indicates that this conclusion does not depend on a uniquely identifiable parameter set.

The combined sensitivity, profile likelihood, and uncertainty quantification analyses demonstrate that, although the individual SEIRD parameters are not practically identifiable from the limited early-stage data, the key dynamical conclusions of the proposed framework remain robust. In particular, the temporal ordering between the inflection point of the susceptible population and the peak of the infected population was preserved across all perturbed parameter realizations. These findings support the central premise of the present work; namely, that the CSP-based analysis extracts robust dynamical information associated with the explosive outbreak dynamics, even in the presence of substantial parameter uncertainty and non-identifiability.

## Appendix D. Forward Uncertainty Propagation and Stability Diagnostics

To address the sensitivity and robustness of the end of the explosive stage, and thus the explosive time scale as a precursor reference, under parameter uncertainty, a forward uncertainty quantification (UQ) framework was deployed to directly quantify the parameter-induced variability of the explosive time scales and local stability thresholds. For each epidemic wave, the optimized baseline parameter vector was subjected to structured perturbations to generate an ensemble of model trajectories. Rather than executing a random Monte Carlo sampling, a multi-dimensional Latin Hypercube Sampling (LHS) strategy was employed, defining a uniform up to ±20% perturbation box centered symmetrically around the nominal calibration values. For each sampled configuration, the governing SEIRD system was integrated numerically from fixed initial conditions over a continuous time horizon to map parameter variations directly to quantitative variations in the underlying CSP metrics.

At each time step, the non-zero eigenvalues and their corresponding time scales were evaluated analytically. The resulting profiles were utilized to construct parameter-induced variability envelopes by extracting the tail quantiles from the empirical distribution of the simulated trajectories at each time coordinate, thereby defining a quantitative sensitivity envelope tracking the forward propagation of parameter variance.

Figure A5 displays these uncertainty bands across all time scales for a uniform ±20% parameter perturbation box. A vertical reference line marks the exact date of the stability transition, i.e., where the explosive mode vanishes, bounded horizontally by a shaded uncertainty band that isolates the temporal variance of this geometric bifurcation point.

For each unique LHS simulation, the exact time point when the explosive dynamics vanish was identified as a core QoI, along with the corresponding value of the explosive time scale at that boundary. Evaluated relative to the unperturbed baseline reference parameters, these structural shifts were expressed as raw temporal and time scale deviations from the nominal reference trajectory to provide a rigorous, non-qualitative sensitivity analysis of the explosive regime’s duration.

The cumulative distributions of these centered variations were compiled into frequency histograms to quantify the statistical variance of the stability boundary. Figure A6 captures the frequency distributions of the transition date shifts, showing whether parameter variations hasten or delay the end of the explosive stage. Symmetrically, Figure A7 illustrates the variations in the critical explosive time scale values at that point. Each figure is centered around the nominal baseline value (zero), highlighted by a primary indicator line to provide a direct, standalone visualization of the sensitivity of the bifurcation threshold.

The resulting diagnostic profiles for all individual wave calibrations are compiled in Figure A5–Figure A7. Notably, in most cases, the envelopes do not expand excessively, demonstrating that the explosive time scale and the corresponding timing of the explosive stage’s end remain robust and exhibit low sensitivity to parameter uncertainty.
